# Experimental Models in Unraveling the Biological Mechanisms of Mushroom-Derived Bioactives against Aging- and Lifestyle-Related Diseases: A Review

**DOI:** 10.3390/nu16162682

**Published:** 2024-08-13

**Authors:** Rajasekharan Sharika, Kuljira Mongkolpobsin, Panthakarn Rangsinth, Mani Iyer Prasanth, Sunita Nilkhet, Paweena Pradniwat, Tewin Tencomnao, Siriporn Chuchawankul

**Affiliations:** 1 Immunomodulation of Natural Products Research Unit, Chulalongkorn University, Bangkok 10330, Thailand; sharikarpillai@gmail.com (R.S.); kuljira.m@chula.ac.th (K.M.); sunitanilkhet1110@gmail.com (S.N.); paweena.p@chula.ac.th (P.P.); 2 Department of Transfusion Medicine and Clinical Microbiology, Faculty of Allied Health Sciences, Chulalongkorn University, Bangkok 10330, Thailand; 3 Department of Pharmacology and Pharmacy, LKS Faculty of Medicine, The University of Hong Kong, Pokfulam, Hong Kong SAR, China; ptkrs@hku.hk; 4 Natural Products for Neuroprotection and Anti-Ageing Research Unit, Chulalongkorn University, Bangkok 10330, Thailand; prasanth.i@chula.ac.th (M.I.P.); tewin.t@chula.ac.th (T.T.); 5 Department of Clinical Chemistry, Faculty of Allied Health Sciences, Chulalongkorn University, Bangkok 10330, Thailand; 6 Department of Clinical Microscopy, Faculty of Allied Health Sciences, Chulalongkorn University, Bangkok 10330, Thailand

**Keywords:** mushrooms, bioactive compounds, *in vitro*, *in vivo*, molecular mechanism

## Abstract

Mushrooms have garnered considerable interest among researchers due to their immense nutritional and therapeutic properties. The presence of biologically active primary and secondary metabolites, which includes several micronutrients, including vitamins, essential minerals, and other dietary fibers, makes them an excellent functional food. Moreover, the dietary inclusion of mushrooms has been reported to reduce the incidence of aging- and lifestyle-related diseases, such as cancer, obesity, and stroke, as well as to provide overall health benefits by promoting immunomodulation, antioxidant activity, and enhancement of gut microbial flora. The multifunctional activities of several mushroom extracts have been evaluated by both *in vitro* and *in vivo* studies using cell lines along with invertebrate and vertebrate model systems to address human diseases and disorders at functional and molecular levels. Although each model has its own strengths as well as lacunas, various studies have generated a plethora of data regarding the regulating players that are modulated in order to provide various protective activities; hence, this review intends to compile and provide an overview of the plausible mechanism of action of mushroom-derived bioactives, which will be helpful in future medicinal explorations.

## 1. Introduction

Mushrooms are abundant and diverse in nature, with an enormous impact on human wellbeing and health. They have been used in medicines and diets by humans worldwide from time immemorable throughout various civilizations [1]. Oriental medicine continuously relies on mushrooms for their various medicinal concoctions. Mushrooms are “macrofungi” that consist of a fruiting body, called a basidiocarp, stipe, and mycelium. They are composed of 90% water and the remaining 10% is accounted for by proteins, amino acids, vitamins, fatty acids, carbohydrates, fiber, ash, and essential minerals [2,3]. Apart from the nutritional value, dietary uptake of mushrooms as a whole or as its derived metabolites, such as polysaccharides, lectins, phenols and polyphenols, ceramides, ergosterols, and terpenoids, which have been identified for their pharmaceutical activities, has shown desirable qualities, such as antioxidant, anti-microbial, anti-inflammatory, antihyperlipidemic, anticancer, anti-allergic, antiviral, immunomodulatory, neuroprotective, and prebiotic properties [1,2,3,4,5,6,7,8,9,10,11,12,13], which makes mushrooms a good choice for novel drug discovery against various diseases associated with oxidative stress, such as cancer, cardiovascular diseases, aging-associated disorders, and diabetes. Hence, there has been an increase in the number of scientific studies aiming to understand the molecular mechanisms underlying the various medicinal properties exhibited by diverse mushroom species.

The past few decades have seen a tremendous increase in mushroom production, owing to the awareness of health and their nutritional benefits. They are currently widely consumed as an alternative to animal protein and products [14]. Among the 16,000 species of mushrooms identified, only 4% are considered to be safe for consumption, although only around 60 species are cultivated commercially [4]. Applying present technological advances to traditional know-how in the elucidation of the structural and biochemical compositions of secondary metabolites isolated from mushrooms has made it possible for the evaluation of their therapeutic properties using *in silico*, *in vitro*, and *in vivo* approaches.

**Figure 1:**
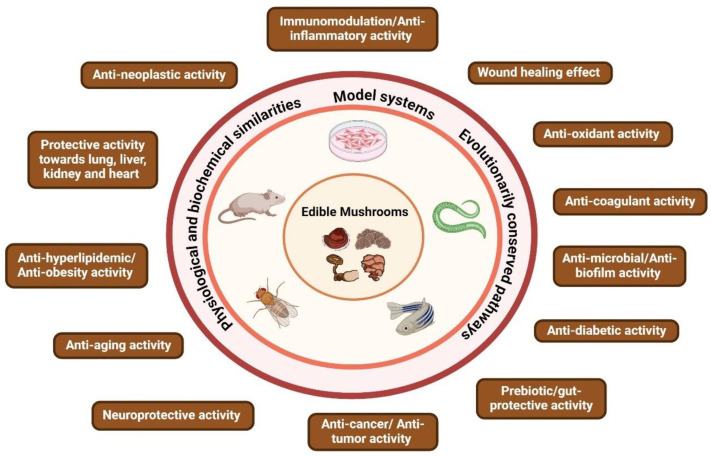
Overview of the different health-enhancing benefits exhibited by edible mushrooms reported in different *in vitro* and *in vivo* models.

The multifunctional activities of several mushroom extracts have been evaluated by both *in vitro* and *in vivo* studies using cell lines along with invertebrate and vertebrate model systems to address human diseases and disorders at functional and molecular levels (Figure 1). Although, each model has its own strengths as well as lacunas, various studies have generated a plethora of data regarding the regulated players that are modulated in order to provide various protective activities; therefore, this review intends to compile and provide an overview of the plausible mechanisms of action of mushroom-derived bioactives, which will be helpful in future medicinal explorations.

## 2. Commonly Explored Models in Mushroom Research

Mushrooms are known to produce large numbers of compounds that are known to have a verity of pharmacological importance in combating or reducing various human pathologies. Model systems also play an important role in assessing the toxicological and pharmacological biokinetics of these therapeutically important mushrooms (extracts and their derived bioactives). Both *in vitro* and *in vivo* models have been used to understand the underlying mechanisms of action and protective effects of the various bioactives, such as polysaccharides, proteins, terpenes, cordycepin, ceramides, and phenolic compounds, in different disease conditions [6,8,12,13,14]. To simplify and distinguish the protective mechanisms of mushrooms and their bioactives, the available reports have been compiled based on the model used for the study and the mode of action involved.

### 2.1. In Vitro Studies

Edible mushrooms are a rich source of antioxidants; therefore, several *in vitro* studies have focused not only on their anticancer and immunomodulatory effects but also on the anti-aging and anti-neurodegenerative effects of their extracts and isolated derivatives. Each mushroom exhibits its own unique modulatory mechanism in various cells lines; therefore, some of the well-explored mushrooms have been tabulated below along with the derivatives and specific cell lines used and modulatory mechanisms (Table 1). Although there are several advantages of *in vitro* systems, including high throughput and cost efficacy, they do not fully mimic the conditions of an organism, thereby lacking to produce a whole picture [15]. Furthermore, several popular mammalian and non-mammalian animals, such a rat, mouse, fish, drosophila, and *Caenorhabditis elegans*, serve as *in vivo* models for human diseases due the presence of either or both physiological or pathophysiological aspects being similar to those of humans. Investigation of several parameters, such as drug efficacy, safety, and toxicological analysis, provide data that could be extrapolated for humans as pre-clinical studies. Furthermore, deep understanding of the molecular mechanism can also be extended to the development of therapeutic drugs against diseases in humans as well as in animals [16].

### 2.2. In Vivo Studies

#### 2.2.1. Mammalian Models

Mouse and rat are the most popularly used animal models due to their close resemblance in physiological and molecular aspects to humans. Both of these animals are social in nature and are efficiently used in understanding various scientific queries regarding nutrition, genetics, immunology, neuroscience, infectious disease, metabolic and lifestyle-related diseases, and behavior. Easy manipulation of physiological conditions through oral or surgical methods or genomic manipulation using CRISPR_Cas9 to manifest desired disease conditions mirroring the human condition is an added advantage [16,150] in analyzing the therapeutic activity of many naturally available bioactives. Table 2 details the various disease models used and the therapeutic as well as regulatory mechanisms of different mushroom derivatives. In mouse models, mushroom derivatives have exhibited antitumor, anti-angiogenic, antidiabetic, anti-allergic, antioxidant, anti-inflammatory, anti-obesity, neuroprotective, wound-healing and gut microbiome protective activities. Similarly, in rats, different mushrooms have been able to modulate tumor growth, activate immunomodulatory activity by regulating the levels of WBC, induce antidiabetic activity by regulating plasma glucose, reduce total cholesterol levels, and enhance learning and memory along with improved neuronal growth, apart from inducing antioxidant effects, which has been explained in detail in Table 2.

#### 2.2.2. Danio Rerio

*Danio rerio* (commonly known as zebrafish) have gained popularity as one of the most suitable non-mammalian vertebrate models for screening potential drug candidates among bioactives and secondary metabolites [269]. The cellular and biochemical similarity between zebrafish and humans have enabled researchers to evaluate the toxicity of small molecules as well as its impact on organs such as the heart, liver, brain, pancreas, and reproductive system. The transparency of the embryo also facilitates studying the interaction between bioactives and regulatory molecules using staining methods or tagging of specific molecular markers [269]. Different mushrooms are able to activate antioxidant mechanisms, reduce lipid accumulation and triglyceride levels, promote larval development and skeletal repair, improve memory, and reduce anxiety, along with reducing the pigmentation and melanin content. The interactions between various mushrooms derivatives and zebrafish have been tabulated below in Table 3.

#### 2.2.3. *Drosophila melanogaster*

*Drosophila melanogaster*, or the fruit fly, has been a valuable tool for understanding the fundamentals of genes and genetic inheritance. Several biochemical pathways that regulate cellular function, as well as about 75% of human disease-related genes, are conserved between human and drosophila. Furthermore, it is easy to manipulate the genetic background to imitate pathogenic conditions to investigate the efficacy of potential drug molecules and targets. Hence, drosophila has been used as an invertebrate model for studying genetic disorders including rare Mendelian genetic disorders, neurodegenerative disorders, immune system regulation, and behavioral studies [286,287]. Various studies have reported that mushrooms can extend the lifespan of both male and female flies, improve AD levels, learning, and memory, as well as regulate apoptosis. They are also known to induce antioxidant activity, and the detailed actions of mushroom derivatives on disease-like conditions studied have been tabulated below in Table 4.

#### 2.2.4. *Caenorhabditis elegans*

**Figure 2:**
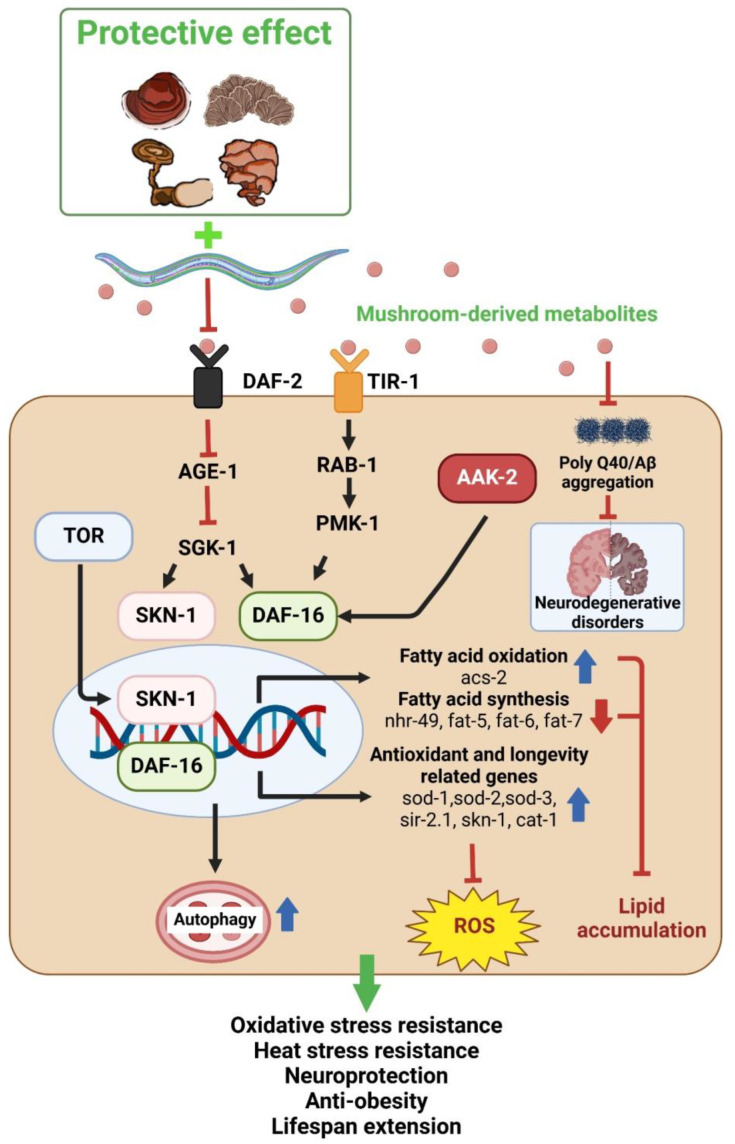
Schematic representation of *C. elegans* as a model to study the overall mechanisms of action of biomolecules isolated from mushrooms. The different biomolecules from mushrooms can modulate different transcription factors, including DAF-16 and SKN-1, regulate stress resistance, and improve anti-aging and neuroprotective potential.

*Caenorhabditis elegans* is another popular invertebrate model in the field of genetic research, which was introduced by Sydney Brenner in 1970’s. Due to the presence of a high degree of homology between *C. elegans* and human pathways, such as insulin signaling, MAPK, and TGF-β pathways, it has been used as a model to investigate various conditions, such as aging, neurodegeneration, inflammation, and immune responses. It is also a promising tool to study the mechanism of action of phyto- and myco-chemicals isolated from natural sources [295] (Figure 2). Mushroom derivatives were reported to extend the lifespan of nematodes under normal as well as stress conditions, which includes oxidative, heat, heavy metal, and high sugar stress. Additionally, mushrooms were able to exhibit antioxidant and neuroprotective effects. All of the effects were modulated by key pathways, which include the DAF-16-mediated pathway, MAPK-mediated pathway, and SKN-1-mediated pathway. The action of mushroom derivatives on *C. elegans* have been tabulated below in Table 5.

#### 2.2.5. Clinical Trials

Clinical trials have been performed on many different medicinal mushrooms, including Agaricus bisporus [309], A. blazei [310,311,312], A. sylvaticus [313,314], Antrodia cinnamomea [315], Coriolus versicolor [316,317], Ganoderma lucidum [318,319], Grifola frondose [320,321,322], Hericium erinaceus [323], Lentinus edodes [324,325,326,327], Phellinus rimosus [328], Pleurotus ostreatus [329], and Poria cocos [330]. Clinical studies in cancer patients undergoing chemotherapy reported that complementary use of mushroom polysaccharides, including lentinal, have improved the survival and quality of life of colorectal cancer patients [331]. A study in Japan also reported the higher response rate of lentinan-administered patients to cancer chemotherapy for solid tumors [332,333,334,335,336,337]. Schizophyllan has been reported to improve the overall survival rate of head and neck-related cancer [338]. Clinical trial reports suggest that schizophyllan consistently improved the overall survival of stage II cervical cancer patients; this, however, was not the case with respect to stage III patients [339,340,341]. Oral intake of *G. frondosa* polysaccharide extracts in 34 postmenopausal breast cancer patients led to a disease-free condition after primary treatment as part of a phase I/II trial, with marked increases in TNF-α, IL-2, and IL-10 production and a one-fifth reduction in IFN-γ production [320]. A single dose of *Hericium erinaceus* in a healthy, young adult cohort resulted in quicker output on the Stroop task at 60 min post dose, thereby improving the speed of performance and reducing subjective stress [323].

A clinical trial on Taiwanofungus camphoratus reported that 8 weeks of oral supplementation of mycelium could make significant reductions in systolic and diastolic blood pressures, probably through interaction with the renin angiotensin system to lower blood pressure by inhibiting renin secretion [342]. Golden *T. camphoratus* administration was also capable of reducing AST, ALT, and TG levels with no significant effects on general safety parameters, suggesting its use as a safe and effective hepatoprotective agent [343]. However, the extract also exhibited negative effects, including gastrointestinal discomfort and a reduction in platelet counts within a month of treatment, even though the role of mushrooms in these effects still remains unclear [315].

Inonotus obliquus exhibited immune-promoting effects, indicating its efficacy in the context of molecular mechanisms of action in immunological disease driven by TNF-α, which pertains to conditions such as psoriasis, along with noticeable relief or complete resolution of gastrointestinal tract symptoms [344,345]. The mushroom was also capable of reducing pain related to peptic ulcers, with more substantial effects seen at higher doses [346]. Tropicoporus linteus regulated immunomodulatory effects by extending natural killer cell activity and elevating IL-6, IgG1, IgG2, and IgM levels in patients with upper respiratory infections [347], reduced knee arthritis symptoms and pain [348], and increased patient adherence to postoperative chemotherapy [349]. However, there is still a wide gap in exploring the role of different mushrooms in human systems, which must be reduced with more clinical trials [350].

## 3. Mushrooms against Aging- and Lifestyle-Related Disorders

### 3.1. Neuroprotective Activity

Neuronal dysfunction comprises neuro-degenerative disorders, neuro-psychiatric disorders, and also neuronal inflammatory disorders. The trigger for the commencement of brain-related dysfunction or disorder can be attributed to genetics, natural aging, an injury, stress, the effect of certain drugs, infection, toxins, as well as other known and unknown factors [351]. Systemic level effects are detected or manifested as either loss of neuronal function, amnesia, gradual or rapid degeneration of neurons, abnormal presence or absence of neurotransmitters, or loss of function of an organ, but they share common cellular and molecular level pathologies, such as the accumulation of misfolded proteins, neuronal inflammation, mitochondrial disfunction, glutamate toxicity, cellular infiltration, and ROS production, which impair neuronal cellular homeostasis [351].

Mushroom-derived nutraceuticals that can stimulate neuroprotective activity in general, as well as disease conditions, via modulation of intrinsic factors are valuable and could transform the present mode of treatment approaches. Several neurodegenerative diseases, including Alzheimer’s and Parkinson’s disease, were found to have reportedly dysfunctional or reduced neuronal growth factors (NGFs) [352]. NGFs play a vital role in the maintenance, differentiation, and survival of the nervous system, as well as maintenance of the cholinergic system [353]. The two unique low-molecular-weight terpenoids, namely, hericenones and erinacines, isolated from the fruiting body and mycelia of *Hericium erinaceus* (HE) reportedly have the ability to cross the blood–brain barrier and have *in vitro* neurotrophic activity via the stimulation of NGFs, are capable of synthesis of NGFs, enhancement of neurite outgrowth in neuroblastoma-glioma cell line NG108-15, as well as enhanced myelination of mature neurons [352,354,355].

Polysaccharide preparations from HE were reported to have free radical inhibitory and scavenging activity by regulating antioxidant enzymes superoxide dismutase, catalase, and glutathione peroxidase, as well as improving the cholinergic system [356,357]. Oral administration of hot water polysaccharide prepared from HE induced enhanced restoration and recovery of damaged peripheral sensory neurons in Sprague–Dawley rats via the Akt and p38 MAPK pathways, comparable to mecobalamin, a drug used for treating traumatic injury that reportedly has gastrointestinal and dermatological side effects [358]. HE polysaccharides were also found to improve damaged visual cognitive memory and spatial short-term memory in an Aβ-induced mouse model [88,359].

The neuroprotective effect of *Ganoderma lucidum* (GL) on spinal cord injury by restoration of GHS, a significant decrease in MDA, the reversal of many histopathological parameters, as well as suppression of inflammatory and oxidative stress has been demonstrated *in vivo* [360,361]. GL polysaccharides were found to suppress neuronal apoptosis, decrease the expression of caspase-3, Bax, and Bim, and increase the expression of Bcl-2, hence enhancing the neuroprotective effect [362,363]. GL aqueous extracts were found to rescue an animal model from hippocampal damage and vasogenic cerebral edema by ameliorating the levels of neurotransmitters and improving neuroplasticity by upregulating CREB/p-CREB/BDNF through the induction of ERK1/ERK2 [364]. Secondary metabolites from the GL fruiting body, methyl ganoderate G1 (a lanostane triterpene), lingzhine E, and lingzhine F (aromatic meroterpenoids), were found to be effective in scavenging Aβ-induced ROS as well as in reducing neurotoxicity, thereby imparting neuroprotectivity [365]. An anti-epileptic effect was observed in both *in vitro* and *in vivo* studies using extracts from GL. Spore-form GL was found to enhance the expression of neurotrophin-4 and stimulation of CaMK IIα in hippocampal neurons while inhibiting n-cadherin expression and accumulation of Ca^2+^. NF-κB expression was also found to be inhibited in the epileptic rat brain. These studies indicate the potential of GL extracts in the protection of neurons and in aiding a therapeutic approach toward decreasing the secondary damage caused by oxidative stress [366,367,368].

Dietary supplementation of *Lentinula edodes* LE β-glucan revealed alleviated neuroinflammation and BDNF deficit as well as synaptic impairment in long-term high-fat diet-fed mice [369]. Methanol extract from the fruiting body found to significantly reduce β-secretase activity [370].

A study by Han et al. [371] demonstrated that *Inonotus obliquus* (IO) polysaccharide administration protected HT22 cells from L-glu-induced damage by improving cell viability through decreasing intracellular ROS accumulation and caspase-3 activity. IO polysaccharide treatment resulted in the restoration of MMP by increasing Bcl-2 levels and suppression of Bax-2. Alleviation of oxidative stress was also observed, as polysaccharide-induced increases in Nrf2 levels were observed. The *in vivo* administration of IO polysaccharide improved AD-like symptoms in APP/PS 1 mice, as revealed by MWM tests, and immunohistopathology also revealed significant reductions in the deposition of Aβ 1-42 and P-Tau. 3,4-Dihydroxybenzalaceton, a catechol-containing compound isolated from IO, showed improved survival of SH-SY5Y cells exposed to PD-related neurotoxin 6 hydroxy dopamine by activating the Nrf/glutathione antioxidant pathway and expression of Akt/PI3K inhibitors [372]. Furthermore, the newly discovered flavan derivative enantiomers and four drimane sesquiterpene lactones isolated from IO also showed enhanced neuroprotective activity in SH-SY5Y cells against H_2_O_2_-induced stress [373].

Interestingly, an aqueous extract of *Trametes veriscolor* reportedly exhibited strong antiradical and AChE inhibition activities in an *in vitro* study [374].

The therapeutic potential of cordycepin, from *Cordysepes* sp., has been widely reported for its anti-inflammatory neuroprotective activity by suppression of the NF κB/AKT/MAPK pathway [375] and pro-inflammatory cytokines, including IL-1β, iNOS, MPOS, and MMP-9, promotion of anti-inflammatory factors [376], inhibition of neutrophil infiltration, amelioration of the BBB, and promotion of anti-inflammatory activity [377]. Oral administration of the low-molecular-weight peptide cordymine increased antioxidant activity, thereby boosting the defense mechanism against cerebral ischemia as well as significantly decreasing cellular infiltration [378].

*Lignosus rhinoceros* (LR) reportedly induced neurite outgrowth in a differentiating Neuro-2a cell line, implicating its neuroprotective activity [116]. *In vivo* analysis showed the reduction of intracellular ROS and a protective effect offered by the ethanol extract of LR on induced oxidative stress by the administration of juglone in wild-type *C. elegans*. LR extracts also reduced the expression of heat shock protein 16.2 and glutathione S transferase GFP protein expression in transgenic *C. elegans* TJ375 and CL2166, respectively. Super dismutase was also found to be enhanced in a dose-dependent manner against the control in CF1553 *C. elegans*. The study demonstrated the activation of antioxidant activity via the DAF-16/FOXO pathway [114]. *In vitro* and *in vivo* neuroprotective activity was offered by the LR extract by alleviating glutamate-induced toxicity in HT22 cells and prevention of neurotoxicity in *C. elegans*, respectively. The supplementation of LR extracts showed enhanced expression of antioxidant genes, as well as the decrease in aggregation of PolyQ40 and improved chemotaxis index in *C. elegans* [308]. A recent study on human embryonic stem cell-derived neuronal lineage cells demonstrated the neuroprotective and neurogenerative activities of LR methanol extract via activation of the Akt signaling pathway [115].

*Sarcodonin* sp. isolates showed significant neurite outgrowth activity in PC12 and NG108-15 cells, a hybrid neuronal cell obtained from mouse neuroblastoma and rat glial cells. The neurite outgrowth induced by SG-ME revealed the PKC-mediated pathway, whereas cyrneine A showed enhanced Rac1 (a small GTPase protein) activity influencing actin dynamics and assembly of F-actin at the tip of the neurite [379,380,381,382,383,384].

Yadav et al. [384] reviewed the neuroprotective efficacy of antroquinonol, a very valuable compound derivative of tetra hydro-ubiquinone isolated from *Antrodia camphorate* (AC). It was shown to improve cognitive skills in APP mouse during oral administration. Allevation of oxidative stress and inflammatory cytokines by stimulating the Nrf2 pathway, which acts through activation of the expression of antioxidant genes and reduction of inflammatory cytokines, which are common pathologies of Alzheimer’s disease. The ability to cross the BBB has made it a plausible choice for bioactives that can influence the neuronal pathway. Another bioactive that can cross the BBB is adenosine, isolated from AC, which can provide neuroprotective activity against neurophysiological disorders, as it binds to the A2A receptor, which results in post-synaptic depolarization.

Uridine, a key isolated bioactive isolated from *Pleurotus gigantus*, induced *in vitro* neurite outgrowth in a dose–time-dependent manner in PC12 cells, regulated via the MEK/ERK and PI3K pathways [385,386].

**Figure 3:**
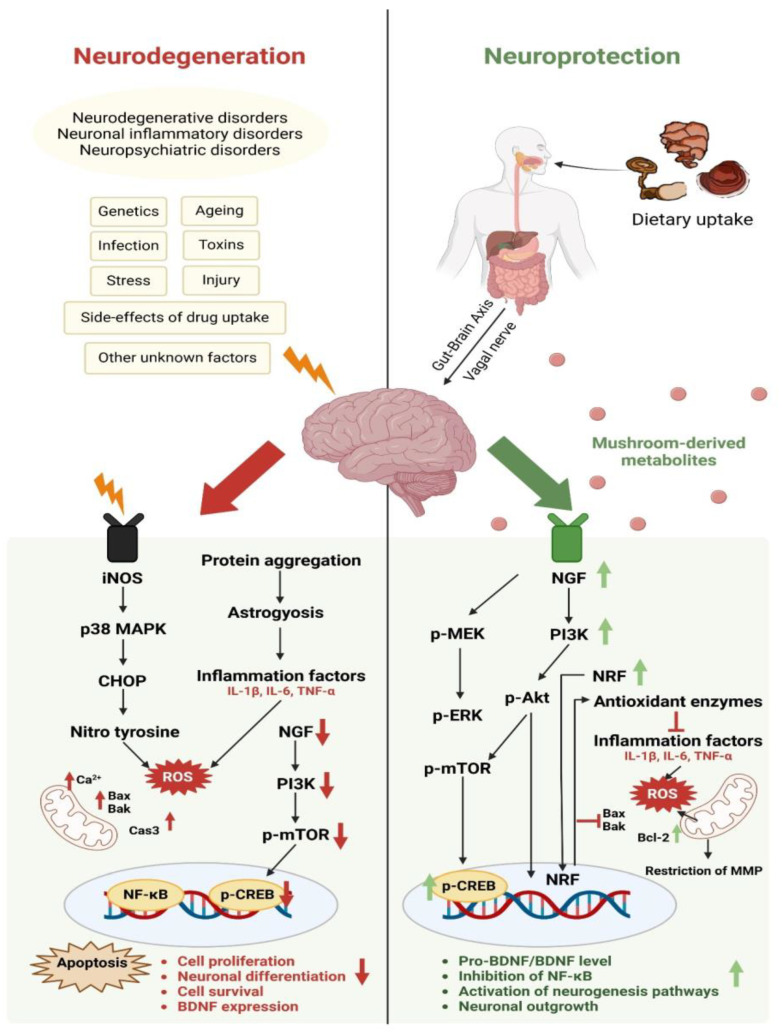
Schematic representation of the impact of dietary inclusion of mushrooms and their protective effects against neurodegenerative, neuropsychiatric, and neuronal inflammation-mediated disorders. Mushroom bioactives are able to improve NRF levels and thereby reduce the levels of ROS and improve neuronal outgrowth.

Inflammation is a double-edged sword, beneficial or damaging depending upon the intensity of its onset. The glial cells, comprising astrocytes and microglial cells, are the main players that regulate the inflammatory response in the CNS. Neuroinflammation is known to be the common pathophysiology seen during neurodegenerating diseases [387,388,389], including Alzheimer’s disease, Parkinson’s disease, Huntington’s disease, epilepsy, brain injury, infection, and other neuropsychiatric disorders, as well as metabolic disorders that can have an impact on the CNS via the gut–brain axis. The various bioactives isolated from mushrooms tend to target neuroimmune modulation to bring about a reduction in the inflammatory response by glial cells and to enhance antioxidant activity both *in vitro* and *in vivo*, thereby promoting neuronal outgrowth and enhancing neuroprotectivity (Figure 3). Most mushrooms have been used in common diets and as dietary supplements; it is important to perform intensive pre-clinical and clinical studies to validate their use as therapeutic agents as neuroprotectants in patients suffering from neurodegenerative disorder.

### 3.2. Antidiabetic Activity

Diabetes is one of the rapidly growing health concerns globally. It is mainly characterized by the increase in blood glucose levels due to insulin deficiency or sensitivity. If neglected, over time it results in defective functioning of the heart, kidney, eyes, blood vessels, liver, and brain, which can result in higher chances of mortality [390]. Several drugs are used in the treatment of diabetes at present, with metformin, a biguanide, being the first line of treatment [391]. Interestingly, guanidine, which is closely related to biguanides, was detected in mushrooms; also, the lectins isolated from *Agaricus* sp. stimulated enhanced insulin secretion from the Islets of Langerhans [392]. Partially pancreatectomized mice were found to have hyperplasia of β-cells along with functional attributes with time of treatment with *Agaricus bisporous* lectins, attributed to the increased expression of D-type cyclin, Cdk4, leading to the hyperphosphorylation of Rb in the islets, resulting in increased PDX-1 and Ngn-3 expression [393].

Aqueous extracts from *Agarius* sp. were found to decrease hyperglycemia and stimulate insulin secretion. Ethyl acetate extract from *A. blazei* was found to induce strong inhibition of α-glucosidase. It was also found to improve glucose uptake in HepG2 cells [394]. Esculetins from *A. bisporous* also were reported to exhibit enhanced α-glucosidase inhibitory activity [395]. Isolates from *Hericium erinaceus* were also found to have α-glucosidase inhibitory activity [396]. α-glucosidase inhibitors are known to suppress the influx of glucose from the intestinal tract to the blood vessels, resulting in a decrease in postprandial glucose levels, hence lowering blood glucose levels [394]. Dietary supplementation of *A. blazei* revealed protective effects against glucose intolerance and insulin resistance in high-fat diet-fed rats [397]. The antidiabetic activity of oligosaccharides isolated from *Agaricus* sp. was found to be twice as potent as that of the β-glucan isolated from the same [398]. *Auricularia* sp. induced modulation of both the antioxidant system and NF-κB signaling pathway as a protective mechanism against diabetes [399]. An interesting study by Chen et al. [400] revealed that the polysaccharides isolated from *A. auricularia* were found to rescue glucose metabolism through modulation of PDX1/GLUT2 in the pancreas and the JNK signaling pathway in the liver of ^60^Co-γ-radiated mice. Similar to α-glucosidase inhibitors, α-amylase inhibitors are also known to reduce postprandial glucose by preventing digestion of polysaccharides, which was observed with *A. polytricha* dietary fibers [401].

Molecules isolated from *Antrodia* sp. were also found to exhibit antidiabetic activity. Antcin K, isolated from *A. camphorata*, improved glucose tolerance in high-fat diet-fed mice by modulating the expression of TNF-α, IL-6, and PPARγ [402]. Antrodin C, isolated from *A. cinnamomea* mycelia, ameliorated hyperosmotic glucose-induced deleterious effects through stimulation of the Nrf-2-dependent cellular antioxidant defense system [403]. *Flammulina velutipes* polysaccharides and isolates from *Inonotus obliquus* were found to be effective in inhibiting diabetic nephropathy progression [404,405]. Active isolates from *Ganoderma* sp. are known to have protective effects against type I and II diabetes mellitus (T1DMand T2DM) as well as other chronic and acute complications associated with diabetes through the regulation of gluconeogenesis and the glycogenolysis process [406]. Alleviation of diabetic neuropathy was observed *in vivo* post oral administration of *Hericium erinaceus* [407], and various studies also revealed the hypoglycemic activity and protective effect of polysaccharides from *Hericium erinaceus* in promoting antidiabetic activity [408]. Polysaccharides extracted from *Trametes versicolor* were found to mitigate the development of diabetic complications as well as to strengthen and reduce the cortical porosity of bines during diabetic conditions [409,410].

### 3.3. Cardiovascular Protection

The most common pathophysiology of cardiovascular diseases is the development of atherosclerosis, primarily due to physical inactivity and high calorie diets. Atherosclerosis is an associated risk factor common among other metabolic disorders, hypertension, and diabetes [411,412]. Several edible varieties of mushrooms have been identified to have antihypercholesterolemic activity, exhibited through the suppression of HMG-CoA reductase [413]. Foods rich in soluble and insoluble dietary fibers have demonstrated efficacy in regulating hyperlipidemia. Mushrooms are rich in β-glucans, which have the ability to modulate the intestinal microbiota as well as influence cholesterol metabolism, lipid absorption, and regulate immunity, along with general health improvement [412,413]. Reduced expression of adhesion molecules (endothelial-leukocyte adhesion molecule-1 [E-selectin], vascular cell adhesion molecule-1 [VCAM-1], and intercellular adhesion molecule-1 [ICAM-1]) and reduced monocyte binding to human aortic endothelial cells are two indications that L-ergothioneine, a diet-derived amino acid, had cardioprotective effects in an *in vitro* atherogenesis model [414].

### 3.4. Cosmeceuticals and Nutricosmetics

Aging is a natural cellular mechanism that affects not only the skin (external appearance) but also the internal organs with the passage of time, though the most striking and noticeable changes occur in the skin as wrinkles, age spots, lack of elasticity, etc. External triggers like pollution and exposure to solar radiation can also result in the production of ROS, causing promotion of collagen breakdown through upregulation of matrix metalloproteinases (MMPs). Biomolecules with effective mechanisms to actively eradicate ROS and reduce the visible changes of aging are being actively researched at present, and mushrooms are one of the most interesting candidates due to their rich antioxidant, anti-inflammatory, antityrosinase, antimicrobial, anticollagenase, antielastase, and antihyaluronidase (moisturizing effect) properties [415]. The various mushroom species that have been traditionally used and commercially marketed have been extensively reviewed by Wu et al. [416] and Srivastava et al. [417].

## 4. Toxicity

There are thousands of mushroom species known to mankind throughout the world, among which a few hundred are edible. However, there are around 150 species reported to be poisonous in the European region, among which some can have a fatal impact [418]. Mushroom toxicity has been determined as a significant cause of concern in modern medicine because a few wild mushrooms that are traditionally consumed, especially in Asia and Eastern Europe, are subject to suspicion due to possible mutagenicity and myotoxicity [419].

The major potentially toxic elements identified in edible mushrooms include metal(loid)s and radionuclides originating from geogenic and anthropogenic sources, which are nonbiodegradable, not easily leachable, with long half-lives. These elements are seen naturally in the environment, and the percentages of these elements are determined by the geology of the region, such as erosion, volcanicity, and weathering [420]. Alpha-emitting radioisotopes are the most toxic among all radionuclides, which are seen in natural and anthropogenic origins in wild mushrooms from around the world. Mushrooms bioaccumulate a range of mineral ionic constituents and radioactive elements to different extents and are therefore considered as suitable bioindicators of environmental pollution. Several natural radionuclides, such as ^210^Po, accumulated at the highest levels were reported in Ukrainian mushrooms, which may expose consumers to highly radiotoxic decay particles produced by alpha emitters [421].

Mushroom foragers consume poisonous mushrooms either out of ignorance or after confusing them with edible ones [418]. However, the toxicity studies performed so far have had contrasting outcomes, leading to vague information about the overall safety of the mushrooms. Added to it, secondary contamination of the mushrooms could also occur during storage or transportation, which also leads to contradictory outcomes regarding consumption [419]. Finally, the limited data on the impact of geochemical anomalies of the soil material on accumulation of toxic elements in mushrooms are hurdles in determining mushroom toxicity.

## 5. Future Perspectives and Conclusions

Edible mushrooms are well known for their immense commercial and medicinal benefits. Mushroom-derived bioactives hold tremendous promise for addressing aging-related and lifestyle-associated diseases, offering a natural and holistic approach to health and wellness. Different innovative healthcare products have been developed from mushrooms, including mushroom coffee, mushroom collagen, mushroom memory enhancers, and many more. Mushroom-based leather can be made, which is durable and biodegradable, as an alternate to animal leather [422]. Mushrooms can be employed to break down and metabolize a wide range of pollutants, including hydrocarbons, pesticides, heavy metals, and even some radioactive materials, thereby aiding in bioremediation [423]. They can also aid in replacing some construction materials, acting as natural binders used in conjunction with more fibrous forms of agricultural waste, such as wood chips, straw, or hemp fibers [424].

The available vast evidence from *in vitro* and *in vivo* studies highlights their multifaceted benefits, ranging from neuroprotection and antidiabetic effects to cardiovascular health and cosmetic applications. The diverse biological activities of these natural compounds make them attractive candidates for developing novel therapeutic and preventive strategies with minimal or negligible side effects. Different mushroom derivatives have reported anticancer, antiviral, antioxidant, anti-inflammatory, antimetastatic, antimicrobial, antidiabetic, neuroprotective, and wound-healing activities in different cell lines, which are explained in detail in Table 1. Future research should focus on the development of more sophisticated *in vitro* models that better mimic the complex interactions within human tissues, which can have immense potential. For instance, 3D cell culture and organ-on-a-chip technologies could provide more accurate insight into how the different bioactives of mushrooms can affect cellular pathways relevant to aging- and lifestyle-related diseases and cancer.

Analyzing the effects of mushrooms in *in vivo* models is essential, as they can mimic the activities of the whole organism. *C. elegans*, being the smallest *in vivo* invertebrate model, highlighted the lifespan extending efficacies of different mushroom derivatives during normal as well as stress conditions. Interestingly, the molecular mechanism could be studied efficiently in this model. Drosophila is another model organism that can provide a better understanding of the effects and regulation of different genes and pathways, as it is a better known model for studying genetics. The zebrafish model, which is a vertebrate and slightly higher than the other models, allows researchers to gain a better idea of the impact on different organs during disease conditions as well as treatment stages. In the case of higher mammalian models, such as mice and rats, the changes in behavior patterns, neurological changes, the impact on aging and age-associated diseases, and the functioning of different internal organs in the presence of mushroom derivatives can be understood. These models have already provided valuable insights into the activities of different mushrooms; however, incorporating more model organisms could aid in uncovering new mechanisms of action and potential therapeutic targets.

Mushrooms can synthesize important biomolecules that are vital to the synthesis of nanoparticles with bioaccumulation and high stability, which is linked to protein capping and amide linkages [425]. Mushroom biomolecules aid in the process of stabilizing and reducing metal ions in nanoparticles, and their negative charges serve as adhesive and electrostatic forces during the formation of nanoparticles [426]. Interestingly, the majority of mushrooms incorporated in nanobiotechnology are edible and highly medicinal, thus serving as biofactories for the production of safe and medically important nanoparticles of iron, silver, selenium, gold, and others [427]. Among all oyster mushroom-mediated nanoparticles, 66% are silver nanoparticles, while 25% are gold nanoparticles and zinc oxide [428]. The biological synthesis of mushroom nanoparticles is stable, environmentally friendly, and non toxic [429].

Mushrooms contain riboflavin, which has light sensitive and water solubility properties that are essential for nanoparticle synthesis. Several biomolecules in mushrooms can reduce and stabilize metal ions in nanoparticles, most especially the biomolecules involved in the transfer of electrons in the complex pathways of NADPH/NADH to NAD^+^/NAD^+^ [430,431,432]. Different factors, including temperature, concentration of the raw materials, pressure, pH of the solution, time, magnitude of the particle, size of the pore, the environment, and accumulation of the mushroom extract can affect the biosynthesis of nanoparticles [433]. The mushroom-sourced biosynthesis of nanoparticles is considered to be safer, biocompatible, ecologically friendly, cost effective, and widely acceptable [429]. For example, resveratrol, a major polyphenol and functional food with poor solubility and bioavailability, was efficiently transported into the host using mushroom-derived nanoparticles [434,435]. The future might lead to a broad range of uses of mushrooms through nanotechnology, especially in the area of biomolecule delivery and tissue biomimetics [429,436].

The integration of omics technologies, such as genomics, proteomics, and metabolomics, into these studies will enhance our understanding of the systemic effects of mushroom bioactives and identify biomarkers for potential activity. Future research should pave the way in that direction, which will be beneficial to mankind. Also, it is important to note that several effects, such as antioxidant, neuroprotective, lifespan extension, and anticancer, were observed in almost every model. This implies that these effects could be conserved in different models and could also be present in humans as well. Even though mushrooms are a part of the human diet in different countries, high-quality human studies have to be designed and performed to confirm the optimum dosage and efficacy of these compounds in diverse populations. Regulatory frameworks must also evolve to accommodate the unique properties of natural products, ensuring that they can be effectively and safely integrated into mainstream healthcare. Future research must therefore focus on integrating innovative models and technologies, conduct comprehensive clinical trials, and thereby explore personalized approaches to therapy. This will allow researchers to better understand the mechanisms underlying their beneficial effects and translate these findings into effective interventions that improve health and quality of life.

## Figures and Tables

**Table 1:** The beneficial activities of various mushroom-derived compounds are summarized based on the various *in vitro* models used and the mechanisms of action.

Mushroom Name	Cell Line Used	Extract/ Compound	Protective Effects	Mechanism	References
*Agaricus blazei* Murill	primary macrophage from rat bone marrow	Aqueous extract	-	Induced macrophage activity via promoting cytokine and NO secretion	[17]
	HEP-2 cells		Antiviral activity against herpes simplex type 1 (HSV-1) and bovine herpes type 1 (BoHV-1)	-	[18]
		Isolated polysaccharide	Antiviral activity against poliovirus infection, Poliovirus type 1-induced infection	-	[19]
	U937, MOLT4, HL60 and K562	Agaritine, β-N-(γ-l(+)-glutamyl)-4-(hydroxymethyl) phenylhydrazine	Antitumor activity	Inhibited tumor cell proliferation	[20]
	Vero cells	Sulfated polysaccharides	Antiviral activity against Herpes simplex virus, HSV-1 KOS and 29 R, and HSV-2 333-induced infection	-	[21]
	MCF-7	Ergosterol	Anticancer activity	Induced cancer cell apoptosis and inhibit cell growth	[22]
*Agaricus bisporus*	LNCaP, PC3, and DU145 cell lines	Conjugated linoleic acid	Anticancer activity	Inhibited prostate cancer cell proliferation	[23]
	Murine J774A.1 macrophages, murine Sarcoma 180 cells, and human cancer cell lines (MCF-7, HT29, DU145 and CRL-1739)	Polysaccharides	Anticancer activity	Induced macrophage activation and inhibit cancer cell growth	[24]
	Caco-2 cells		Anti-inflammatory and antioxidant activity, LPS- and TNF-α-induced inflammation	-	[25]
	THP-1 cells		Immunomodulatory effect	Activation of pro-inflammatory cytokine production	[26]
*Auricularia auricula-judae*	A549 cells	Lectin (Ammonium-sulfate fractionation)	Antitumor	Modulation of JUN, TLR4, and MYD88 expression	[27]
	HeLa cells	Chloroform extract	Anticancer activity	-	[28]
	HCT-15, huh-7, SK-MEL-5, SNU-213, and SNU-484	Polysaccharide	Anticancer activity	Anticancer cell proliferation and anti-oxidative enzyme. Inhibition of the peroxiredoxin 1 pathway	[29]
	Ba/F3 TPR-Met and Ba/F3 TPR-TrkB cell lines	Ethyl acetate extract	Anticancer activity	Inhibition of cancer cell growth via suppressing tropomyosin receptor kinase B activity. Obstruction of auto-phosphorylation of TrkB	[30]
	HepG2 cells	*Auricularia auricula* polysaccharide-3-1	Antioxidant activity	Reduction of reactive oxygen species production, malondialdehyde, and increased activities of superoxide dismutase, glutathione peroxidase, and catalase	[31]
*Auricularia polytricha*	A549 and 3T3-L1 cells	Polysaccharides (Ethanol extraction)	Anticancer activity	Inhibition of cancer cell proliferation and induced cell apoptosis. Increased p53 and p21 levels and decreased cyclin A, cyclin D, and CDK2 expression	[32]
	BV2 and HT-22 cells	Ergosterol (Hexane and ethanol extraction)	Anti-inflammatory and antioxidant activities	Increased SOD-1 expression and modulation of the NF-κB signaling pathway in BPA-induced inflammation	[33,34]
*Antrodia camphorate*	MCF-7 and HBL100 cells	Fermented culture broth	Anticancer activity	Inhibition of cell viability, chromatin condensation, inter-nucleosomal DNA fragmentation, and sub-G1 phase accumulation	[35]
	MDA-MB-231 cells		Antimetastatic activities	Inhibition of MMP-9, MMP-2, uPA, uPA receptor (uPAR), and vascular endothelial growth factor (VEGF) and suppression of phosphorylation of ERK1/2, p38, and JNK1/2	[36]
	HER-2/Neu-overexpressing SKOV-3 cells and human ovarian surface epithelial (IOSE) cells		Anticancer activity	Inhibition of HER-2/Neu activity, tyrosine phosphorylation, activation of PI3K/Akt and their downstream effector β-catenin	[37]
	RAW 264 cells (LPS induced)		Anti-inflammatory activity	Reduced tumor necrosis factor (TNF-α) and interleukin (IL)-1β levels and decreased cytokine, iNOS, and COX-2 expression by blocking NF-κB activation	[37]
	HT-29 cells	Five lanostanes (2, 3, 4, 6, and 8) and three ergostane types (1, 5, and 7) triterpenes. (Chloroform extraction)	Anticancer activity	Induced cytotoxicity and sub-G1 cell cycle arrest	[38]
	MDA-MB-23, A549, and HS68 cells	Twelve ergostanoids, named antcamphins A–L (1–12), together with 20 known triterpenoids. (95% ethanol extraction).	Anticancer activity	Induction of cytotoxicity	[39]
	MCF-7, MDA-MB-231, MCF10A, and HS-68 cells	Antrocin	Anticancer activity	Induction of cleavage of caspase-3 and poly (ADP-ribose) polymerase, reduced Bcl-2 and Bcl-xL, and suppressed phosphorylation of Akt and the activities of its downstream effectors mTOR, GSK-3β, and NF-κB	[40]
	CL1-0, CL1-5, A549, H1975, H441, PC9, and BEAS-2B cells		Anticancer activity	Increased expression of caspase-3, increased Bax/Bcl2 ratio, and downregulated the JAK/STAT signaling pathway	[41]
	HTB-4 cell line	Phosphate-buffered saline (PBS) at the ratio of 1:25 (*w*/*v*)	Anticancer and anti-metastatic activities	Inhibited MMP-9 and induced phase G2M arrest	[42]
	Huh7, HepG2, and Hep3B cell lines	Antcin A, antcin C, and methyl antcinate A	Anticancer activity	Induced sub-G1 population, DNA fragmentation, TUNEL-positive cells, and caspase activation	[43]
	LNCaP, PC-3, and MEF cell lines	Ethanol extraction	Anticancer activity	Induced G1/S phase arrest, inhibited cyclin D1 activity, and prevented pRb phosphorylation	[44]
	MCF7, T47D, MDA-MB-231, MCF10A, and IMR-90 cell lines	Antroquinonol D	Anticancer activity	Inhibited DNMT1 methyltransferase activity	[45]
	Human erythrocytes	Aqueous extraction	Antioxidant activity	Increased glutathione (GSH) and ATP levels in peroxyl radical [2,2′-Azobis(2-amidinopropane) dihydrochloride, AAPH]-induced oxidation	[46]
	COLO 205 cell line	5-Substituted 4,7-dimethoxy-1,3-benzodioxoles (compounds 1–9) (Ethyl acetate extraction)	Anticancer activity	Induced G0/G1 cell cycle arrest and increased p53, p21, and p27 levels	[47]
	PC12 cell line (6-hydroxydopamine-induced)	95% ethanol extraction	Neuroprotective	Reduced the loss of dopaminergic neurons. Increased tyrosine hydroxylase (TH) and dopamine transporter (DAT) levels and reduced α-synuclein levels	[48]
*Boletus edulis*	LS180 and CCD 841 CoTr cell lines	Biopolymers (polysaccharides and glycoproteins)	Anticancer activity	Induced G0/G1-phase arrest and inhibited the p16/cyclin D1/CDK4-6/pRb pathway	[49]
		Ribonucleic acid	Anticancer activity	Increased Bax, TP53, and CDKN1A levels	[50]
	MDA- MB-231 and Ca761	Cold water-soluble polysaccharide (BEP); galactose, glucose, xylose, mannose, glucuronic, and galacturonic acid	Anticancer activity	Induced Bax/Bcl-2 ratios, the release of cytochrome C, and activated the expression of caspase-3 and caspase-9	[51]
	MCF-7, SMMC-7721,hHL-60, SW480, and A549 cells	Non-isoprenoid botryane sesquiterpenoids, named boledulins A-C (1–3)	Anticancer activity	-	[52]
	MCF-7, HepG-2, CaCo, CFPAC, Hela, U-87, HT-29, SK-MEL-28, and A549 cells	*Boletus edulis* lectin (BEL) β-trefoil (Aqueous extraction)	Antineoplastic properties	-	[53]
	BEL-7402, HT-299, SPC-A1, and U-251 cells	5-Cholestene-2,3-oxide, β-sitosterol, and stigmasterol	Anticancer activity	Induced cytotoxicity	[54]
	MCF7, HT-29, HUH-7, and L929 cells	Aqueous extraction	Anticancer, antimicrobial, and wound-healing activities	-	[55]
	RAW264.7 cells	Polysaccharide (BEP)	Immunomodulatory activity	Induced phagocytosis and NO, IL-6, and TNF-α secretion	[56]
Flammulina velutipes	A549	Fungal immunomodulatory protein-five (FIP-fve)	Anticancer activity	Induced p53 and p21 expression and inhibited EGF-induced activation of Rac1 via decreasing RACGAP1 mRNA and protein levels	[57]
	MCF7 and MDA-MB231 cells	5% ethanol extraction	Anticancer activity	Induced DNA damage (γ-H2AX foci formation), G2/M phase arrest, cytochrome c release, and caspase cleavage activity	[58]
	BGC-823 and A549 cells	Polysaccharide (FVP-1 and FVP-2) (Ultrasonic-assisted extraction)	Anticancer activity and antiproliferative activity	-	[59]
	MCF-10a, MCF-7, and MDA-MB-23 cells	Aqueous and methanol extraction	Antiproliferative and antioxidant activities	-	[60]
	RAW264.7, L929, and B16F10 cells	Polysaccharide (FVSP-1, FVSP-2 and FVSP-3) (Ultrasonic-assisted extraction)	Anticancer activity and antiproliferative activity	-	[61]
	SGC and LoVo cells	Ergosterol and 22,23-dihydroergosterol		Induced cytotoxicity	[62]
	HeLa and LS174 cells	Ethanol extraction	Antioxidant activity	-	[63]
	HL-60, HeLa, rc ts-NRK, and FM3A cells.	Enokipodins A, B, C, and D	Anticancer activity	-	[64]
	L929 cells	Polysaccharides	Antioxidant activity	Reduced reactive oxygen species (ROS) production	[65]
	HepG2 and L02 cells		Anticancer activity	Induced ER stress via increasing intracellular Ca2+ concentrations by activating the phospholipase C–inositol-1,4,5-triphosphate (PLC-IP3) pathway	[66]
	K562 cells	FVPA1 (a novel polysaccharide)	Immunomodulatory activity	Induced natural killer cell activity	[67]
	PC12 cells (H_2_O_2_ induced)	Flavonoids (arbutin, epicatechin, phillyrin, apigenin, kaempferol, and formononetin)	Antioxidant properties	Increased cell viability, glutathione levels, and superoxide dismutase activity	[68]
*Ganoderma lucidum*	MDA-MB-231 and PC-3 cells	Aqueous extraction	Anticancer activity	Induced AP-1 and NF-κB expression and inhibited urokinase-type plasminogen activator (uPA) expression	[69]
	IOSE-398, OV2008, C13*, A2780s, A2780-cp, and SKOV-3 cell line.		Anticancer activity	Induced cell cycle arrest at the G2/M phase and activated caspase-3 and p53 levels	[70]
	MCF-7 cell line	ethanol extraction	Anticancer activity	Induced cell cycle arrest and apoptosis via increasing p21/Waf1 and decreasing cyclin D1 levels	[71]
	SW 480 cell line	polysaccharides and triterpenoid	Anticancer activity and antioxidant activity	Inhibited cell proliferation	[72]
	HUC-PC model	95% ethanol extraction		Induced cell apoptosis, inhibited telomerase activity, and increased oxidative stress	[73]
	95-D, SMMC7721, KB-A-1, KB-3-1, and HeLa cells and human normal cell lines HLF and L-02	Ganoderic acid T (GA-T), a lanostane triterpenoid	Anticancer activity	Induced cell cycle arrest at the G1 phase and increased p53 and Bax levels, while decreasing the Bcl-2/Bax ratio	[74]
	RAW264.7 cells	Triterpene	Anti-inflammatory activity	Decreased tumor necrosis factor-α (TNF-α), interleukin-6 (IL-6), inflammatory mediator nitric oxide (NO), and prostaglandin E2 (PGE2) levels	[75]
	BV-2 cells	Ganoderenic acid C, ganoderic acid C2, ganoderic acid G, ganoderenic acid B, ganoderic acid B, ganoderic acid A, ganoderic acid H, ganoderenic acid D, ganoderic acid D, ganoderic acid F, and polysaccharides	Anti-inflammatory activity	Decreased G-CSF, IL1-α, MCP-5, and MIP3α, with a higher effect on MIP3α levels, and reduced mRNA expression of CHUK, NFκB1/p150, and IKBKE (NFƙB signaling)	[76]
	RAW 264.7 cell line, inflamed colonic biopsy specimens of patients with Crohn’s disease and Human Peripheral Blood Mononuclear Cells	Triterpene ganoderic acid C1 (GAC1)	-	Reduced TNF-α, IFN-γ, and IL-17A production and inhibited the NF-κB signaling pathway	[77]
	PC12 (corticosterone-induced) and RAW264.7 cells (LPS induced)	Aromatic compounds (lucidumins A-D and lucidimine E)	Neuroprotective and anti-inflammatory activities	-	[78]
	RAW264.7 cells	Triterpenes (butyl lucidenateE2, butyl lucidenateD2 (GT-2), butyl lucidenate P, butyl lucidenate Q, Ganoderiol F, methyl ganodenate J, and butyl lucidenate N)	Anti-inflammatory activity	Induced HO-1 expression via the PI3K/AKT-Nrf2 pathway and decreased tumor necrosis factor-α, interleukin-6, nitric oxide synthase, and cyclooxygenase-2 expression	[79]
		Ergostane-type steroids, C28 steroids	Anti-inflammatory activity	Reduced nitric oxide production	[80]
	HaCaT cells	Ganoderic acids	Antioxidant, antidiabetic, and anti-inflammatory activities	Decreased the response to inflammation-related cytokines at the mRNA level	[81]
*Ganoderma resinaceum*	BV-2 cells	Lanostane triterpenoids (ganoresinoids A-D) and meroterpenoid (ganoresinoid E)	Anti-inflammatory, antioxidant, and anti-apoptosis activities	Reduced nitric oxide (NO), IL-1β, IL-6, and TNF-α levels and inhibited the TLR-4/NF-κB and MAPK signaling pathways	[82]
*Ganoderma duripora*	RAW 264.7 cells	Farnesyl phenolic compounds, ganoduriporols A and B	Anti-inflammatory activity	Inhibited tumor necrosis factor-α (TNF-α), interleukin-1β (IL-1β), interleukin-6 (IL-6), and prostaglandin E2 (PGE2) through suppression of the COX-2, MAPK, and NF-κB signaling pathways	[83]
*Ganoderma sinense*		Ergosterols, ganocalidophins A–C (1–3)	Anti-inflammatory activity	Inhibited NO production	[84]
*Hericium erinaceus*	Chago-K1	Peptides	Anticancer activity	Scavenged free radicals, induced apoptosis, and increased caspase-3, -8, and -9 levels	[85]
	FHC and HCT-116 cell lines	Polysaccharide	Anticancer activity	Inhibited the growth of colon cancer cells and induced S phase cell arrest	[86]
	RAW264.7 cell line		Immunomodulatory effects	Increased NO, IL-6, and TNF-α production	[87]
	PC12 cell line		Antioxidant and neuroprotective effects	Reduced ROS production and increased mitochondrial membrane potentials in amyloid beta-induced neurotoxicity	[88]
	GES-1 cell line		Antioxidant activity	Induced cell proliferation, inhibited cell necrosis, reduced ROS levels, regulated mitochondrial membrane potential, and maintained mitochondrial membrane permeability in H2O2-induced oxidative damage	[89]
	MCF-7 cell line	Aqueous extraction	Anticancer activity	Induced cell apoptosis and G1 cell cycle arrest and changed the expression of a total of 362 transcripts	[90]
	GES-1 and MC cell lines	Polysaccharide (EP-1)	Anticancer activity	Induced cell apoptosis and cell cycle arrest at the G0/G1 phase and regulated Bax, Bcl-2, and caspase-3 levels	[91]
	SGC-7901 cell line	Polysaccharide-protein HEG-5	Anticancer activity	Reduced Bcl2, PI3K, and AKT1 levels and increased caspase-8, caspase-3, p53, CDK4, Bax, and Bad expression	[92]
	EA hy926 cell line	Ethanol extraction	Anticancer activity	Inhibition of antiangiogenic and antioxidant potentials and regulated the MMP-9/NF-κB and Nrf2-antioxidant signaling pathways	[93]
	U937 cell line	Hot water (HWE), microwaved 50% ethanol (MWE), acidic (ACE), and alkaline (AKE) extracts	Anticancer activity	Induced the activation of mitochondria-mediated caspase-3 and caspase-9	[94]
	K562, LANCAP, and HEP2 cell lines	Diterpene, 4-chloro-3,5-dimethoxybenzyl alcohol, 7α, 8β, 11-trihydroxydrimane, 3-acetyl-4-methoylbenzoic acid, 1-(5-chloro-2-hydroxyphenyl)-3-methyl-1-butanone, and 4-chloro-3, 5-dimethoxybenzoic acid	Antibacterial activity and anticancer activity	Inhibited the growth of *Helicobacter pylori*	[95]
	U87 cell line	Erinacerin O and erinacerin P, novel glioma inhibitors	Antitumor activity	Induced the apoptosis rate, reduced DNA replication, and regulated the Bax/capase-3 pathway	[96]
	SH-SY5Y, 1321N1, Caco-2, HCT-116, OVK18, and HeLa cells	Isoindolin-1-ones, named erinacerins (M–N)	Anticancer activity	-	[97]
*Inonotus obliquus*	B16–F10 cell line	Water extract	Antiproliferation	G_0_/G_1_ cell cycle arrest. Reduction of cyclin E/D1 and Cdk 2/4 expression	[98]
	3LL cell line	Hot water extract	Anticancer activity	-	[99]
	HT-29 cell line		Antitumor activity	Induced apoptosis, inhibited tumor growth by regulation of Bcl-2, Bax, and caspase-3	[100]
	A549, H1264, H1299, and Calu cell lines	Chagabusone A and triterpenoids	Anticancer activity	-	[101]
	PC3 and MDA-MB-231 cell lines	Ethyl acetate	Antiproliferation	-	[102]
	RINm5F cells	Polysaccharides	Antioxidant activity	Inhibited insulin secretion, regulation of caspase-3, Bax, Bcl-2, NF-κB, and MAPKs	[103]
	HepG2 cells		Hypoglycemic activity	-	[104]
	SMMC7721, and Jurkat cell lines		Antitumor activity	-	[105,106]
*Lentinula edodes*	MRC-5 and A549 cell lines	Polysaccharides: branched α-(1,4)-glucan (L10	-	Induced THP-1 differentiation, increased superoxide and interleukin-8, RANTES production, decreased angiogenin and vascular endothelial growth factor, regulation of TLR-4/MyD88/ IKK/NFkB	[107]
*Lignosus rhinoceros*	ORL-204 cell line	High-molecular-mass fraction (HMM)	Anti-oral cancer activity	Modulated tumor necrosis factor (TNF) cell signaling leading to apoptosis, proliferation inhibition (cell cycle arrest), and immunomodulation	[108]
	MCF-7 and A549 cell line		Antiproliferative activity	-	[109]
	MCF-7 cell line	Protein fraction termed F5	Anti-breast cancer activity	Increased extrinsic and intrinsic apoptotic pathways	[110]
	PC12 cell line	Peptides (Thr-Leu-Ala-Pro-Thr-Phe-Leu-Ser-Ser-Leu-Gly-Pro-Cys-Leu-Leu) (Crude protein extract)	Neuroprotective effects	Neuroprotective effects against 6-hydroxydopamine-induced toxicity. Enhanced cellular antioxidant activity and inhibited nuclear factor-kappa B (NF-κB) activation	[111]
		Water and ethanol extracts	Cytoprotective effects	Cytoprotective effects against hydrogen peroxide (H_2_O_2_)-induced oxidative stress. Decreased apoptosis via inhibition of caspase-3/7 activities	[112]
		Hot water and ethanol extracts, and isolated polysaccharide	Neuroprotective effects	Neurite outgrowth stimulatory effect. Upregulated the MEK/ERK1/2 signaling pathway	[113]
	HT22 cell line	Ethanol extract	Antioxidant activity	Protective effects against glutamate-induced toxicity Increased antioxidant-related genes, including catalase (CAT), superoxide dismutase (SOD1 and SOD2), and glutathione peroxidase (GPx)	[114]
	(hESC)-derived neural lineages	Methanol extract	-	Protection against dexamethasone (DEX)-induced toxicity. Inhibited apoptosis and reduction of phospho-Akt (pAkt) levels	[115]
	Neuro-2a and PC-12 cell line	Water extract	-	Neurite outgrowth stimulatory effect	[116,117]
	NHDF cell line	F5 thermoresponsive gel	Wound-healing activity	-	[118]
*Pleurotus citrinopileatus*	Caco-2 cells	Sphingolipids	-	Colon injury suppression. Reduced LPS-induced cell death by inhibiting apoptosis-related proteins	[119]
	HepG2 and HeLa cell lines	Methanol extract	Antitumor activity	Induced apoptosis and cell cycle arrested at the G1 and G2/M phases	[120]
	HepG2 cell line	Crude polysaccharide-peptides	Hepatoprotection	Reduced high-fat diet-induced hepatocyte injury by inhibiting lipid accumulation and enhancing SOD enzymatic activity and the adiponectin pathway	[121]
	U937 cell line	A non-lectin glycoprotein	Antitumor activity and immune modulation	Induced a shift of T helper cells toward Th 1 response by activating TNF-R, IFN-γ, and IL-2 secretion and indirect inhibition of cell growth	[122]
	K562, HCT116, DU-145, and PC-3 cell lines	Sesquiterpenoids	Antidiabetic and antitumor activities.	Reduced cancer cell viability and inhibited the protein tyrosine phosphatase 1B enzymatic activity	[123]
*Pleurotus eryngii*	Ana-1 cell line	Adenosine	Immunomodulatory activity	Increased the proliferative rate of Aha-1 cells and elevated TNF-α and IL-6 levels	[124]
	U937 cell line	Ubiquinone-9	Antitumor activity	Induced apoptosis by inhibiting DNA topoisomerase I activity	[125]
	3T3-L1 cell line	Chloroform extract	Anti-adipogenic activity	Lipid accumulation was inhibited in adipocytes by targeting PI3K/Akt/mTOR signaling, which resulted in PPAR γ and C/EBP suppression	[126]
	RBL-2H3 cell line	Ethanol extract	Allergic prevention and anti-inflammation activity	Decreased pro-inflammatory cytokines and histamine levels by inhibiting NFAT-, NF-кB-, and FceRI-mediated signaling	[127]
	PBMCs and U937 cell lines	Powder	Immunomodulatory activity	Increased activated Th2 cells, dendritic cells, and macrophages in PBMCs and regulated TNF-α and IL-10 levels	[128]
	RAW 264.7 cell line	Polysaccharides	Immunomodulatory activity	Produced nitric oxide, TNF-α, IL-1, and IL-6 through the p38, JNK, and MAPK signaling pathways	[129]
	HepG2 cell line		Antitumor activity	-	[130]
				Hypoglycemic activity. Restored insulin resistance by activation of the PI3K-Akt signaling pathway	[131]
	Mouse-derived dendritic cells		Immunomodulatory activity	Immunomodulation by increasing the production of nitric oxide and TNF-α levels. Cytokines were activated through TLR2/TLR6 and dectin-1 receptors induced by β-glucan	[132]
	A549, BGC-823, HepG2, HGC-27, and RAW264.7 cell lines	Protein fractions	Antitumor activity and immunostimulant activity	Induced cytotoxicity against cancer cells and stimulated lysosome activity, pinocytosis, and nitric oxide production in RAW264.7 cells with no toxicity against normal liver cells	[133]
*Pleurotus ostreatus*	MCF-7 cell line	Silver nanoparticles (Biomass)	Antitumor activity	Inhibition of cell growth	[134]
	MDA-MB-231, MCF-7, MCF-10A, HCT-116, HT-29, and FHC cell lines	Methanol extract	Antitumor activity	Inhibited the cell cycle at the G0/G1 phase of breast and colon cancer cells via p53-dependent as well as p53-independent pathways	[135]
	PC12 cell line	Polysaccharides	Neuroprotective effects	Ameliorated PC12 cells from H2O2-induced oxidative damage through the PI3K/Akt signaling pathway and inhibited apoptosis-related pathway proteins	[136]
	Vero, MCF-7, HepG2, CaCo-2, and HeLa cell lines	Polar extract	Antitumor activity	Induced cell cycle arrest in the sub-G1 stage and apoptosis. Expression of TNF-α was increased while IL-6 expression was decreased	[137]
	RAW264.7 cell line	Ultrasonication and heating	Anti-inflammation activity	Inhibited nitric oxide and TNF-α production in LPS-induced inflammation macrophages	[138]
		Water extract	Anti-inflammation activity	Suppressed LPS-induced secretion of tumor necrosis factor-a (TNF-a, interleukin-6 (IL-6), and IL-12p40	[139]
		Selenium-enriched polysaccharides	Immunomodulation	Induced macrophages to produce proinflammatory cytokines (NO, ROS, TNF-α, IL-1β, and IL-6) by activating the NF-κB pathway	[140]
	HepG2, MCF-7, SKOV3, HeLa, and PC-3, L02, MCF-10A, and IOSE cell lines		Antitumor activity	Induced apoptosis by disrupting the Bax/Bcl-2 protein ratio and inhibiting the epithelial-to-mesenchymal transition	[141]
	SW480 cell line	Water-soluble protein extract	Antitumor activity	Induced apoptosis in cells partially through ROS production, GSH depletion, and mitochondrial dysfunction	[142]
*Pleurotus pulmonarius*	Huh7, Hep3B, and SMMC-7721 cell lines	Polysaccharide-protein complex	Antitumor activity	Inhibited cell growth, colony formation, and invasion by suppressing the PI3K-Akt signaling pathway	[143]
*Trametes versicolor*	HeLa and Jukart cell lines	PSK	Antiproliferation	G_0_/G_1_ cell cycle arrest, direct cytotoxicity. Increased lymphocyte proliferation (synergistic with IL-2)	[144]
	AGS, A549, B16, and Ando-2 cell lines		-	Induced apoptosis by regulating caspase-3	
	HeLa	Cold buffer extract	Anticancer, antiproliferation	-	[145]
	4T1 cell line	Polysaccharides	Antitumor, Antimetastasis, Immunomodulation	-	[146]
	MOLT4 cell line	PSP	Antitumor	Inhibited cancer cell growth, S phase arrest, and induced apoptosis	[147]
	PBMC cell line		-	Increased proliferation, IL-1β, TNF-α, and IFN-γ	
			-	Increased monocyte numbers by possible regulation of TLR2/6/4 and Dectin-1	[148]
	LN-CaP cell line	Ethanol extract	Antiproliferation	Reduced secretion of prostate-specific antigen (PSA) in an androgen receptor-independent manner	[149]
	PC-3 and DU-145 cell lines		Modest antiproliferation		

**Table 2:** The beneficial activities of various mushroom-derived compounds are summarized based on the mammalian model used and the mechanism of action.

Mammalian Model	Mushroom Name	Extract/ Compound	Protective Effects	Mechanism	References
Mice	*Agaricus blazei* Murill	1,6-beta-glucan	Antitumor activity in sarcoma 180-bearing ICR mice	-	[151]
	*Agaricus brasiliensis*	Sodium pyroglutamate	Anti-angiogenic and antimetastatic activity	Inhibited tumor growth. Increased CD8+ T and natural killer cell levels and von Willebrand factor expression	[152]
		β-glucan	Antitumor activity	Inhibited tumor growth, reduced IL-10 levels, and induced IFN-gamma production	[153]
		Hot water and cold water extracts	Antitumor activity	Antitumor effect, and increases in CD4+ T and natural killer cells were observed. Reductions in cholesterol levels and blood glucose levels were also observed in sarcoma 180-bearing ICR mice and LPS and Concanavalin A-induced inflammation and hepatic injury, respectively, in Balb/c mice	[154]
		Aqueous extraction	-	Prevention of allergy via reducing cytokine levels. Reduction of anti-OVA Ig-E levels and Th2 relative to Th1 cytokine levels in ovalbumin-induced allergy in NIH/OlaHsd, C57Bl/6, and BALB/c mice	[155]
		Fucogalactan	-	Antinociceptive activity in acetic acid-induced abdominal contraction in male Swiss mice	[156]
		Agarol (an ergosterol derivative	Anticancer activity	Induced cancer cell apoptosis and anti-tumor activity. Increased ROS production, AIF levels, and Bax levels, and decreased Bcl-2 levels in SCID mice	[157]
	*Agaricus bisporus*	Aqueous extraction	Antitumor activity	Inhibited tumor growth and increased tumor cell apoptosis in BALB/c Nu-Nu athymic mice	[23]
			Antitumor activity	Inhibited tumor growth and induction of nuclear factor-κB with the production p50/105 heterodimers in male C57BL/6 murine sarcoma model (injected 180 cells)	[24]
			Anticancer activity	Inhibition of cancer cell proliferation via suppression of androgen receptor and cell cycle progression in male intact C57BL mice and tumor-derived xenograft/PDX tumor-implanted male NSG intact mice.	[158]
	*Auricularia auricula-judae*	Auricularia auricula judae polysaccharide-cisplatin complex (AAP-CDDP)	Anticancer activity	Upregulation of Bax, cytochrome-c, and caspase-3, downregulated Bcl-2 expression, and induced superoxide dismutase, catalase, and glutathione peroxidase activities. A decrease in mitochondria potential was also observed. (HeLa (cervical cancer) cell line-induced cancer in female BALB/c mice).	[159]
		β-D-glucan	Anticancer activity	Induction of apoptosis through upregulated Bax and downregulated Bcl-2 expression. (Sarcoma 180 (S-180) tumor cell-induced cancer in male BALB/c mice).	[160]
		Aqueous extraction	-	Enriched the arginine biosynthesis pathway. Changed the gut microbiota composition. (C57BL/6J male mice)	[161]
		Melanin	-	Inhibition of CYP2E1 and activation of Nrf 2 along with its downstream antioxidase. Reduction of ALT, AST, TG, and MDA levels and increased antioxidant enzyme levels, such as ADH, SOD, and CAT. (50% ethanol-induced acute alcoholic liver in male C57BL/6 mice)	[162]
		Polysaccharide (hot water and ultrasonic-assisted extraction)	Anticholestrimic effect	Reduction of serum total cholesterol and low-density lipoprotein cholesterol levels. Increased total antioxidant capacity and lipoprotein lipase activity. (Cholesterol-enriched diet-fed male ICR mice)	[163]
		Polysaccharide (methanol extraction)	-	Significantly accelerated wound closure through fibroblast and keratinocyte proliferation, migration, and invasion. Promotion of collagen synthesis and reduction of E-cadherin expression	[164]
	*Auricularia cornea* var. Li. (an evolutionary varieties of A. auricula-judae)	Polysaccharide	-	Inhibition of aldose reductase and CYP2E1 as well as protein expression of iNOS and COX-2 in ADL mice model. Furthermore, expression of pro-inflammatory players, such as IL-1β, TNF-α, and IL-6, was found to be decreased, and SOD, GSH-Px, and CAT were found to be modulated.	[165]
	*Auricularia polytricha*	Polysaccharides (APPIIA)	Antitumor activity	Antitumor effect and induced macrophage activation. (180 cells injected in male BALB/c albino mice)	[166]
		Polysaccharides	-	Decreased Bax and caspase-3 expression, increased Bcl-2 expression, and anti-fibrosis effect. Protection against chronic kidney diseases.	[167]
		95% ethanol extraction	Hepatoprotective activity	Modulation of ALT and AST activities, impeded the TLR4/NF-κB and caspase signaling pathways, and induced the Keap1/Nrf2 signaling pathway to promote a hepatoprotective effect in dextran sulfate sodium-induced ulcerative colitis in specific pathogen-free ICR male mice.	[168]
	*Antrodia camphorate*	Fermented culture broth	-	Decreased tumor size. Reduction of cell proliferation markers such as cyclin D1 and PCNA. Reduction of Bcl-2, which resulted in the promotion of apoptosis.	[169]
		Phosphate-buffered saline (PBS) at the ratio of 1:25 (*w*/*v*)	Hepatoprotective activity	Reduction of plasma aspartate aminotransferase (GOT) and alanine aminotransferase (GPT) levels and induced the activities of SOD, glutathione, and catalase in CCl4-induced liver toxicity in male ICR mice.	[170]
		4,7-Dimethoxy-5-methyl-1,3-benzodioxole	Anticancer activity	Induced p53-mediated p27/Kip1 protein and reduced cyclin D1, D3, and A levels in COLO-205 human colon cancer cell-injected BALB/c nu/nu mouse.	[171]
		Ergostatrien-7,9(11),22-trien-3β-ol	-	Reduction of p65NF-κB and caspase-3 expression, induced PI3K/Akt, as well as inhibited GSK-3 levels in transient focal cerebral ischemia-induced male ICR mice	[172]
	*Boletus edulis*	Polysaccharide	Antitumor activity	Real (BUN and creatin) and liver (AST and ALT) damage parameters were found to be significantly reduced in treated mice. Acted as a mitogen in tumor-bearing mice. Induction of antitumor activity through cytotoxic lymphocytes (NK and CTL cells). Increased secretion of IL-2 and TNF-α.	[173]
			Anti-asthmatic	Restoration of lung pathology, reduced IL-4 and IFN-γ levels, and increased CD4+CD25+FOXP3+ Treg cells in ovalbumin-induced asthma in female BALB/c mice.	[174]
		Isolated protein (BEAP)	Antitumor activity	Significantly decreased PARP and caspases-3, -8, and -9. Increased the Bax/Bcl-2 ratio, implying a tumor reduction through induction of apoptosis in A549 cell-injected female BALB/c nude mice.	[175]
		Fungal nitrite reductase	-	Inhibited nitrite-induced toxicity by reducing nitrite in blood, hence increasing the lifespan of sodium nitrite toxicity-induced male Kunming mice	[176]
		A water-soluble polysaccharide (BEBP); BEBP-1, BEBP-2, and BEBP-3	-	Increased SOD and decreased MDA levels in the serum of Kunming mice with D-galactose-induced oxidation.	[177]
	*Flammulina velutipes*	Starch-free β-type glycosidic polysaccharide	Gut protection	Reduction of morphological and physiological changes in the colon. Reduction of pro-inflammatory cytokines such as TNF-α, IL-6, MCP-1, and MIP-1α and increased the relative expression of tight junction proteins such as claudin-1, occludin, and zonula occludens. Dramatic change in gut microbiota was also observed in dextran sulfate sodium (DSS)-induced colitis in C57BL/6J male mice.	[178]
		Polysaccharide/polysaccharides consisting of glucose linked with β-glycosidic bonds.	Gut protection	Induced production of short-chain fatty acids. Improved gut microbiota through immunomodulation of expression of TNF-α, IF-γ, IL-6, and IL-8.	[179,180]
		Sulfated polysaccharides (SFPS)	Anti-aging	Decreased levels of ALT, AST, and ALP (liver toxicity index) as well as CRE, BUN, and UA (kidney toxicity index). Improved lipid metabolism and resisted aging as well as organ damage induced by d-galactose in male Kunming strain mice.	[181]
		Polysaccharides	Neuroprotective activity	Reduction of IL-1β, TNF-α, IL-6, and IL-10 levels and decreased escape latency and total swimming distance in scopolamine-induced learning and memory impairment in male mice (C57BL/6).	[180]
			Gut protection	Protective effect by activation of the Akt/GSK3β/Nrf-2/HO-1 signaling pathway and modulation of gut microbiota against Pb-induced toxicity in SPF-grade Kunming male mice.	[182]
			Antidiabetic activity	Modulation of the PI3K/Akt signaling pathway to reduce blood glucose and insulin levels and regulate dyslipidemia.	[183]
			Hepatoprotective activity	Hepatoprotection through the reduction of AST, ALT, triglyceride (TG), total cholesterol (TC), and total bile acid (TBA) contents, change in liver histopathology, and decrease in IL-6, IL-1β, and TNF-α levels in carbon tetrachloride-induced hepatic oxidative injury in male C57BL/6 mice.	[184]
		Immunomodulatory protein (FVE)	Antitumor activity	Increased survival and inhibition of tumor size and angiogenesis through regulation of INF-γ in BNL hepatoma cell-injected female BALB/c mice. Increased MHC class I and II and costimulatory CD80 molecules on peripheral blood mononuclear cells.	[185]
	*Ganoderma applanatum*	Terpenes	Hepatoprotective activity	Reduced Cu/Zn-SOD, CAT, GPx, and GST activities, decreased IL-1β and COX-2, and inhibited NF-κB translocation in male Kunming strain mice with benzo(α)pyren-induced oxidative stress and inflammation, hence providing anti-inflammatory activity, antioxidant activity, and hepatoprotective effects.	[186]
	*Ganoderma cochlear*	(±)-Dispirocochlearoids A–C (1–3), meroterpenoids with a 6/6/5/6/6/6 ring system	Anti-inflammatory activity	Inhibition of neutrophil and macrophage infiltration, decreased protein concentrations in bronchoalveolar lavage fluid, inhibition of COX-2 in lung tissue, and suppression of PEG2 and proinflammatory cytokines in LPS-induced acute lung injury mice.	[187]
	*Ganoderma tsugae*		-	Inhibition of allergic airway by reducing leukocyte influx, eotaxin levels, histamine levels, and PGE_2_. No significant reductions in NO and proinflammatory cytokines (IL-1β and IL-6) were observed in ovalbumin (OVA)-induced allergic asthma in female BALB/c mice.	[188]
	*Ganoderma lucidum*	95% ethanol extraction	-	Reduction of E-cadherin, mTOR, eIF4G, and p70S6K and activation of extracellular regulated kinase (ERK1/2) levels in SUM-149 cell-injected female severe combined immunodeficient (SCID) mice.	[189]
		Aqueous extraction	Antitumor activity	Decreased tumor growth and volume. Regulation of NAG-1. Relative expression of P16 and RB1 (retinoblastoma gene) was found to be significantly increased. Protein mRNA expression of FOXO3a was found to be increased, also the mRNA level of P21 was found to be increased. WEE1and E2F1 mRNA expression was found to be significantly reduced as well as reductions in cyclin D1 and B, PCNA, and Ki67. Dose-dependent reductions in anti-apoptotic gene expression, such as Bcl-2, NF-κβ, and c-FOS, were also observed in HCT116-injected male BALB/C nude mice.	[190]
		Ganodermanontriol, a lanostanoid triterpene	Antitumor activity	Tumor volume and weight were found to be reduced and immunohistochemistry revealed a significant reduction in cyclin D1 in HT-29 colon cancer cell-injected male nude immunodeficient mice (nu/nu).	[191]
		Triterpene acids (lucidenic acids and ganoderic acids) and Sterols (fungisterol, 5,6-dihydroergosterol, ergosterol, ergosterolperoxide, 9(11)-dehydroergosterol peroxide, and demethylincisterolA3)	Anti-inflammatory and antitumor activity	Anti-inflammatory and antitumor-promoting effects in TPA-induced ear-edema inflammation in specific pathogen-free female ICR mice and 7,12-dimethylbenzene[a]anthracene (DMBA) and TPA-induced two-stage mouse skin carcinogenesis in SENCAR mice.	[192]
		Beta 1,3/1,6 glucan	-	Significant production of IgA in serum	[193]
		Ethanol extract	Anti-inflammatory	Reduction of malondialdehyde levels. Inhibition of acute and chronic inflammation induced by carrageenan and formalin, respectively, in Swiss albino mice	[194]
		Ganoderic acid A and ergosterol	Neuroprotective activity	Promoted motor performance and protected against loss of dopaminergic neuronal cells in MPTP-treated mice (Parkinson’s model). Protective effect through the MPK/mTOR/ULK1 and PINK1/Parkin pathways.	[195]
	*Hericium erinaceus*	Aqueous extraction	-	The extracts were found to be more active and less toxic compared to 5-FU against HT-29, NCI-87, and Huh-7 xenografts and also significantly delayed tumor doubling time. It showed similar effects in a dose-dependent manner against HepG2 xenograft in SCID mice. Reduction of tumor growth, increased NO, elevated phagocytic activity, and angiogenesis was also significantly inhibited.	[196]
			Antitumor activity	Reduction of tumors. Increased NK cell activity and elevated phagocytic activity of macrophages. Inhibition of angiogenesis by inhibiting VEGF, COX-1, 5-LOX, PGE_2_, and LTB_4_ in CT-26 colon cancer cell-transplanted in BALB/c mice.	[197]
		Hot water and microwaved 50% ethanol extracts	Antitumor activity	Histopathological evidence revealed reductions in tumor nodules and metastasis in lungs. Inhibition of metastasis by decreasing matrix metalloproteinases MMP-2, MMP-9, extracellular signal-regulated kinase (ERK), c-Jun N-terminal kinase (JNK), and p38 mitogen-activated protein kinase (MAPK) phosphorylation in CT-26 colon cancer cells transplanted in pathogen-free female BALB/c mice.	[197]
		Polysaccharides	-	Ameliorated diarrhea, rectal bleeding, reduction in weight, and colitis in 2% DSS (*w*/*v*)-induced colitis in male C57BL/6 mice. Suppressed MDA and myeloperoxidase activities, improved T-SOD levels, and IL-β, TNF-α, and IL-6 activities were greatly reduced through the attenuation of NF-κB, AKT, and MAPK phosphorylation. iNOS and COX-2 were found to be decreased in a dose-dependent manner.	[198]
		Erinacine A	Antiproliferative effect	Antiproliferative effect observed in DLD-1 cell-injected BALB/c-nu mice could be attributed to the cell cycle G1 arrest induced by the compound in DLD 1 cells (*in vitro*). ROS, JNK, 1/2MAPK, and mTOR pathways were also found to be involved.	[199]
		Nine selenium polysaccharide derivatives, sHEP1-sHEP9	Immunomodulatory activity	Induced DC maturation and increased MHC-II and CD86, phosphorylation of ERK, p38, and JNK, and the nuclear translocation of transcription factors p-c-Jun, p-CREB, and c-Fos in LPS-induced ICR mouse.	[200]
		70% ethanol extract	Neuroprotective activity	Significant hippocampal neuroprotection post acute seizures in pilocarpine-induced status epilepticus in male C57BL/6 mice. Increased NeuN-expressing cells. Reduced COX-2-expressing cells.	[201]
	*Inonotus obliquus*	Polysaccharides	Antitumor activity	Tumor growth was found to be reduced 3-fold in treated (ip post-administration) in BALB/c mice. The effect could be attributed to G_0_/G_1_ phase cell arrest and activation of caspase-3-mediated apoptosis as observed in *in vitro* analysis. Proteins p53, pRb, and p27 were found to be decreased in a dose-dependent manner.	[98]
			Anti-diabetic and Nephroprotective activity	Significantly reduced fasting blood glucose levels, insulin tolerance, triglyceride levels, and elevated HDL/LDL ratio. Urine albumin-creatin levels were found to be decreased. NF-κB and TGF-β levels were found to be decreased in a dose-dependent manner in HFD/STZ-induced nephropathy in C57BL/6 mice	[202]
			Anti-diabetic activity	IgA, TNF-α, and IL-6 were found to be significantly reduced in the intestine region. H&E staining showed improvement in histological structures of intestine tissue in extract-treated diabetic mice. The mRNA expression of Ki-67, ZO-1, and MUC2 genes was found to be significantly upregulated in a dose-dependent manner.	[203]
			Hepatoprotective activity	ALT and AST were found to be decreased, serum SOD and liver GHS levels were found to be increased, and NO levels were found to be decreased. Serum levels of TNF-α, IFN-γ, IL-4, IL-6, and IL-1β were found to be decreased. Alleviated hepatocyte enlargement, cytoplasmic vacuolation, cellular infiltration, and necrosis. TLR-2, TLR-4, and pNF-κBp65 were found to be suppressed and p-IκBα was significantly downregulated. Nrf2 and HO-1 expression was found to be increased.	[204]
			Antitumor and anticancer activity	Tumor growth regression. Enhancement of T and B cells. Anti-cancer mechanism by promoting cytokines IL-2, IL-6, IL-12, and TNF-α. Stimulation of macrophage function. Promoted apoptosis via promoting Bax-2 and inhibiting Bcl-2 expression in a concentration-dependent manner in Jurkat tumor-bearing Kunming mice.	[106]
			-	Metabolic regulation, reduced blood glucose, and improved lipid metabolism. Improved serum profiling and reversed metabolites leucine and proline.	[205]
			Antidiabetic activity	Effectively prevented loss of body weight, controlled blood glucose, and increased insulin sensitivity. Alleviated oxidative stress induced by hyperglycemia. The PI3K/Akt pathway plays a role in modulating metabolism in high-fat diet- and STZ-induced T2DM mice.	[206]
			Immunomodulatory activity	Restoration of damaged colon by alleviating colon tissue injury and tight junction protein deficiency. Possible modulation of Th17-, Th2-, Th1-, Treg-related cytokines through the JAK-STAT (p-STAT1, p-STAT6, p-STAT3) signaling pathway in colitis-induced mice.	[207]
		Hot water extract	Antitumor activity	Decreased tumor progression, vascularization, and metastasis. Maintenance of optimum body temperature.	[99]
		Trp-Gly-Cys tripeptide	-	Platelet aggregation inhibitor	[208]
		Ethanol extract	Anticancer activity	Increased expression of p53, p21, Bax, and caspase-9 was observed. EBV viral proteins BZLF-1 (key factor for EVB lysis reactivation) and LMP-2 (essential for EVB latency) were found to be moderately repressed in NOD/SCID mice implanted with EBV+ human gastric carcinoma (SNU719).	[209]
	*Lentinula edodes*	Mycelia extract (hot water)	-	Lowered the increased TGF-β and IL-6 plasma levels in C57BL⁄6 (H-2b) BALB⁄c Nu⁄Nu (H-2 d) mice inoculated with C26. Inhibition of Th17 cells and myeloid-derived suppressor cells.	[210]
		Mycelia extract	Antitumor activity	T cell-dependent antitumor activity. Decreased Tregs and TGF-β	[211]
	*Lignosus rhinocerus*	Hot water extract	Antiasthmatic activity	Inhibition of airway hyperresponsiveness (AHR) in asthma model in house dust mite (HDM)-induced asthma in BALB/c mice.	[212]
			Antiasthmatic activity	Inhibition of airway inflammation in asthma model. Attenuated IgE, Th2 cytokines, CD4+ T cell population, leukocyte infiltration, and mucus-producing goblet cells in the lung epithelium	[213]
		Rhinoprolycan fraction	Antitumor activity	Airway relaxation effects. Antitumor activity in MCF7-xenograft NCr nude mice.	[214]
		Polysaccharides	Immunomodulatory activity	Inhibition of immunosuppressive activity. Improved immune organs and stimulated the release of cytokines TNF-α and INF-γ in cyclophosphamide (Cy)-induced SPF Kunming mice.	[215]
	*Pleurotus citrinopileatus*	Polysaccharides	Antitumor activity	Decreased tumor size and lowered the mortality ratio in ICR mice with Sarcoma 180 tumor-bearing.	[216]
			Immunomodulatory activity	Increased Nrf2, Keap1, p62, HO-1, and NQO1 expression in immunocompromised mice via the p62/Keap1/Nrf2 signaling pathway. The immune activity was activated by phagocytic activity, Th1/Th2 balance, and cytokine production in male SPF-grade Kunming mice.	[217]
			Antitumor activity	Tumor weights were reduced and apoptosis increased. Cell cycle arrest at the S phase. The immunity of tumor-bearing mice was improved in the spleen index with polysaccharide treatment in male mice bearing H22 hepatoma tumors.	[218]
			Anti-obesity activity	Improved body weight, lipid accumulation, and serum biochemistry parameters in high-fat diet-induced obese (DIO) C57BL/6J mice.	[219]
		Lectin	Antitumor activity	The tumor size was reduced and anti-HIV-1 reverse transcriptase was inhibited in mice bearing Sarcoma 180 tumors.	[220]
		Lipid fraction	-	Reduced morphological changes in the colon and reduced inflammation stress in DSS-induced chorionic crypt injury in male BALB/c mice.	[119]
		Water extract	Anti-inflammatory activity	Alcoholic steatohepatitis prevention. Decreased serum lipid profiles, cellular lipid accumulation, and inflammation by activating the SIRT1–AMPK and P2X7R–NLRP3 inflammasome in ethanol-induced male C57BL/6 mice.	[221]
	*Pleurotus eryngii*	Ethanol extract	-	Reduced lipid absorption and carbohydrate-degrading enzyme activity (α-amylase) in male C57BL/JJmsSlc mice.	[222]
		Polypeptides	Immunomodulatory activity	Restored the complication from cyclophosphamide-induced immunosuppression by shifting the thymus/spleen index, lymphocyte count, and gut microbiota abundances in male Kunming strain mice.	[223]
			Antitumor activity	Inhibited the tumor volume in mice and enhanced NK cell and spleen activities with higher TNF-α and IL-2 serum levels in female BALB/c mice with renal-bearing cancer.	[224]
		β-type glycosidic polysaccharides	Anticolitis activity and probiotic enhancement	Inhibited proinflammatory cytokines (TNF-α, INF-γ, and IL-10) via NF-κB and improved gut microbiota in dextran sodium sulfate (DSS)-induced colitis in male CD-1 (ICR) mice.	[225]
		Water extract	Immunomodulatory activity	Gut and liver immunity were improved by regulating NrF2, Nfkb, DNMT1, and IL-22 genes in CD1 mice and whole peripheral blood and fecal samples collected from healthy donors.	[226]
			Anti-obesity activity	Lipid absorption was inhibited by reducing pancreatic lipase enzymatic activity in high-fat diet-fed C57BL/6 male mice.	[227]
		Heterogalactan	Immunomodulatory activity	Macrophages were activated by p38, JNK, and NF-κB via TLR2 and splenocyte activation was induced by the TLR4-PKC axis in cyclophosphamide (CTX)-immunocompromised BALB-C mice.	[228]
	*Pleurotus ostreatus*	Glucans	Antitumor activity	Activated nitric oxide production in macrophages and inhibited tumor growth in Swiss albino mice with Sarcoma 180 (S-180) tumors.	[229]
		Polypeptides	Immunomodulatory activity	Immune cell populations (T cells, NK cells, macrophages) were increased, activating the gut microbiota to balance system immunity in female C57BL/6 mice.	[140]
		Dried powder	Anticancer activity	Abolished the effect of BBN in mice by stimulating NK cell and lymphocyte activities in female ICR mice with BBN-induced carcinogenesis.	[230]
			Anti-obesity activity	Improved body weight, serum lipids, blood sugar, and liver and kidney functions in high-fat diet-fed C57BL/6J male mice	[231]
		Ethanol extract	Antidiabetic activity	Reduced body weight and serum lipid profiles and increased HDL cholesterol levels and antidiabetic activity in alloxan-induced diabetic BALB/C mice.	[232]
			Anticancer activity	Prevented colon injury and carcinogenesis via suppression of COX-2, F4/80, Ki-67, and cyclin D1 in PhIP-induced male ICR mice.	[233]
		Proteoglycans	Anticancer activity	Inhibited cell growth and arrest at the G0/G1 phase. Stimulated NK cells and macrophage functions in Swiss albino mice with Sarcoma 180 (S-180) tumors.	[234]
		Water extract	Immunomodulatory activity	Immune regulation and malnutrition relief through increased total liver proteins and DNA and protein contents in gut mucosa and stimulated humoral immunity Balb/C mice.	[235]
			Immunomodulatory activity	Suppressed secretion of TNF-α and IL-6 in LPS-induced inflammation and inhibited interferon-g (IFN-g), IL-2, and IL-6 in concanavalin A (ConA)-stimulated mouse splenocytes in BALB/c mice.	[139]
	*Pleurotus pulmonarius*	Dried powder	Allergic relief	Showed no effect on antigen-induced nasal rubbing and sneezing and inhibited histamine release from rat mast cells in female BALB/c mice.	[236]
		b-glucan-rich fraction	Memory improvement	Suppressed histological changes and neuronal loss and reduced neuroinflammation by reducing Iba-1-positive microglial cells in high-fat diet-fed female ICR mice.	[237]
		polysaccharide-protein complex	Antitumor activity	Suppressed the VEGF-induced PI3K/AKT signaling pathway in liver cancer cells in Huh7 tumor-bearing male BALB/c mice.	[143]
		Glucan	Colitis prevention	Improved colon damage, decreased MPO activity levels, and decreased proinflammatory cytokines (IL-1b and TNF-α) in DSS-induced colitis female BALB/c mice.	[238]
			Colitis prevention	Inhibited cell proliferation, induced apoptosis, and inhibited inflammation in DSS-induced colitis FVB/N mice.	[239]
	*Trametes versicolor*	Polysaccharopeptide (PSP in combination with IL-2	Antitumor activity	Decreased ROS production. Both early and late treatment induced IL-2 expression. An increase in TNF-α and reduction in TGF-β was observed in tumor cells in BALB/cByJ mice with H238.	[240]
		Polysaccharides	Antitumor activity	Significant decrease in tumor weight. Preservation of bone integrity (CT images). Increases in IL-2, -6, and -12 but no change in IL-10. Increases in IFN-γ and TNF-α in BALB/c mice bearing 4T1 tumors.	[146]
			Immunomodulatory activity	Activation of splenocytes and selective binding to CD19^+^ cells and CD14^+^ cells. Ig class switching and IL-2 production were observed. TLR4-mediated B cell activation, p38 MAPK pathway activation, and cytosolic translocation of NF-κB p65 were observed in female BALB/c, C3H/HeJ, and C3H/HeN mice.	[241]
		Aqueous extract in combination with metronomic zoledronic acid (mZOL)	Antitumor activity	Diminished tumor growth, protected bones, and inhibited metastasis in the liver and lungs in female BALB/c Nu/Nu nude mice inoculated with MDA-MB-231-TXSA.	[242]
		PSP	-	Slightly increased glutathione S-transferase (GST) activity and increased blood GPX activity. Possibly P450-mediated metabolism in male C57 mice.	[243]
Rat	*Agaricus blazei* Murill, *Agaricus brasiliensis*	Aqueous, acid, and alkaline extraction	Antitumor activity	Inhibited tumor growth and increased body weight. Increased liver catalase and superoxide dismutase activities in Walker-256 tumor-bearing rats.	[244]
	*Agaricus bisporus*	Powdered mushroom	Antidiabetic activity	Reduction of plasma glucose and cholesterol (LDL). No change in triglyceride levels and protected against hepatic toxicity in streptozotocin-induced type 2 diabetes in male Sprague–Dawley rats.	[24]
	*Auricularia auricula-judae*	Aqueous extract	Antidiabetic activity	Reduced plasma glucose, total cholesterol, triglyceride, GOT, and GPT levels in streptozotocin-induced diabetic male Sprague–Dawley (SD) rats.	[245]
			Immunomodulatory activity	Promoted immune modulatory effect by inducing total and differential WBCs in cyclophosphamide-induced immunodeficiency in Wistar rats.	[246]
		Hot water and ultrasonic-assisted extraction	-	Reduction of bronchoalveolar lavage fluid and lung edema, significantly inhibited myeloperoxidase (MPO) activity and malondialdehyde (MDA) levels, decreased TNF-α and IL-6 in the blood, and alleviation of LPS-induced pathological changes in the lungs of LPS-induced inflammation in adult Sprague–Dawley rats.	[247]
	*Auricularia polytricha*	Aqueous extract	Hepatoprotective activity	Reduced AST, ALT, ALP, LDH, TB, TG, and cholesterol levels and increased total protein levels. Hepatoprotective activity in paracetamol-induced liver toxicity in Sprague–Dawley rats.	[248]
			Anticholestremic activity	Decreased total cholesterol and LDL and increased HDL levels in reused cooking oil-induced hyperlipidemia in Wistar rats	[249]
		Aqueous extract- soluble polysaccharide	Antihyperlipidemic effect	Decreased total cholesterol and LDL and increased HDL levels in high-fat diet-induced hypercholesterolemia in male Sprague–Dawley (SD) rats	[250]
	*Antrodia camphorate*	Oral treatment	-	Suppression of iNOS and HO-1 expression and reduction of Bax and caspase-3 in thromboembolic cerebral tissue. Inhibition of OH• signals was also observed.	[251]
		Water extract and ethanol extract	Memory improvement	Enhanced long-term and short-term memory and learning ability. Significantly reduced ROS levels in hippocampus as well as p-tau and Aβ40 in Aβ-infused male Wistar rats	[252]
	*Flammulina velutipes*	Aqueous extraction	Neuroprotective activity	Induction of cell proliferation and elongation, stimulated nerve functional recovery and axonal outgrowth, and increased growth-associated protein 43 (GAP-43) and the JAK2/STAT3 pathway in female Sprague–Dawley rats	[253]
		Enokitake (*Flammulina velutipes*) fiber	-	Reduced LDL (VLDL), intermediate-density lipoprotein (IDL), and LDL-cholesterol concentrations in cholesterol-free diet with cellulose powder-fed male F344/DuCrj rats.	[254]
	*Ganoderma atrum*	polysaccharide	Anti-inflammatory and antioxidant activity	Reduction of 8-OHdG levels, increased superoxide dismutase (SOD), catalase (CAT), and glutathione peroxidase (GSH-Px) activities and IL-10 levels, and prevented the overproduction of malondialdehyde (MDA), IL-1β, IL-6, and TNF-α in male SD rats with acrylamide-induced inflammation and oxidative damage.	[255]
	*Ganoderma microsporum*	immunomodulatory protein	Antioxidant activity	Reduction of oxidative damage and cognitive impairment. Increased superoxide dismutase 1 (SOD-1) and lowered astroglia proliferation in traumatic brain injury-induced female and male Sprague–Dawley rats	[256]
	*Ganoderma lucidum*		Neuroprotective activity	Neuroprotective effect, reduction of the cerebellar infarct area, neurological functional deficits, neuronal apoptosis, and decreased active caspase-3, -8, and -9 and Bax levels in middle cerebral artery occlusion (MCAO) in Sprague–Dawley (SD) rats and oxygen and glucose deprivation (OGD) in primary cultured rat cortical neurons	[257]
	*Lignosus rhinocerus*	Hot water extract	Anti-asthmatic activity	Inhibition of airway inflammation in asthma model. Attenuated IgE, Th2 cytokines, leukocyte infiltration, and mucus-producing goblet cells in the lung epithelium of Sprague–Dawley rats with ovalbumin (OVA)-induced asthma.	[212]
		High-molecular-mass fraction (HMM)	-	Airway relaxation effects. Suppressed carbachol-, 5-hydroxytrptamine-, and calcium-induced airway contractions in Sprague–Dawley rats.	[258]
		Cold water extract	-	Bronchodilator effect mediated by the calcium signaling pathway downstream of Gαq-coupled protein receptors in Sprague– Dawley rats.	[259]
		Freeze-dried mushroom powder	Antioxidant activity	Antioxidant activity and amelioration of diabetic complications in streptozotocin-induced diabetic rats.	[260]
		High-molecular-mass fraction (HMM)	Anti-inflammatory activity	Anti-acute inflammatory activity in carrageenan-induced paw edema in Sprague–Dawley rats.	[261]
	*Pleurotus citrinopileatus*	Ethyl acetate and methanol extract	-	Reduced serum cholesterol and triglycerides in high-fat diet-fed mice and increased glutathione peroxidase and superoxide dismutase activities in the blood of female hamster rats.	[262]
	*Pleurotus eryngii*	Methanol extract	-	Estrogen-like activity and bone loss prevention. Trabecular bone density was increased in the extract treatment group in eleven-week-old female Sprague–Dawley rats (240–260 g) with removed ovaries.	[263]
		Cellulose	Hepatoprotective activity	Fatty liver prevention. Reduced ALT, AST, TC, and TG levels in the serum of high-fat diet-fed rats and decreased fatty accumulation in the liver in male SD rats	[264]
		Chitin	Cardioprotective activity	Serum lipid levels decreased and ALT, AST, and SOD enzymatic activities improved. Prevented liver steatosis and aortic atherosclerosis in male Sprague–Dawley (SD) rats.	[265]
	*Pleurotus ostreatus*	Water-soluble polysaccharide	Gastroprotective effect	Inhibited oxidative stress from acetic acid-induced gastric lesions and increased mucus synthesis.	[266]
	*Pleurotus pulmonarius*	Metabolites	Antitumor and immunomodulatory effects	Suppressed leukemia induction and elevated the phagocytic index of macrophages in leukemia-induced Wister rats.	[267]
		Mycelial hot water extract and (HWE) and acetone extracts (AE)	Antidiabetic activity	Reduced serum lipid profiles and elevated high-density lipoprotein cholesterol in diabetes-induced Wistar albino rats.	[268]

**Table 3:** The beneficial activities of various mushroom-derived compounds are summarized based on the zebrafish model used and the mechanism of action.

Model	Mushroom Name	Extract/ Compound	Protective Effects/ Mechanism	References
*Danio rerio*	*Agaricus bisporus*	Powdered mushroom + *Lactobacillus casei*	Upregulated the expression of growth-related (*gh* and *igf1*), mucosal immune-related (*tnf-α*, *lyz*, and *il1b*), and antioxidant-related (*sod* and *cat*) genes.	[270]
		β-glucan	Reduced lipid accumulation and triglyceride levels. Lowered C/EBP α, c SREBP1, LXR α, PPAR γ, and increased LC3 II/LC3 I. Induction of autophagy.	[271]
		Aqueous extract + *Lactobacillus acidophilus* and *Lactobacillus delbrueckii* subsp. *Bulgaricus*	Acted as a prebiotic to *Lactobacillus acidophilus* (La) and *Lactobacillus delbrueckii* subsp. *Bulgaricus* (Lb). Feeding La and Lb improved growth and reproduction, as well as increased *cyp19a* gene expression.	[272]
		Glucosamine hydrochloride	Promoted larval skeletal development and caudal fin regeneration and increased the expression of bone specific markers such as *col1a2*, *col10a1a*, and *col2a1a*. Promoted skeletal injury repair in osteoporosis model of zebrafish larvae and adults. Activation of Bmp signaling.	[273]
		Ethanol extract	Inhibited melanogenesis	[274]
	*Antrodia cinnamomea*	Ethanol extracts	Reduced the melanin content and inhibited tyrosinase activity.	[253]
	*Ganoderma lucidum*	Deacetyl ganoderic acid F	Attenuated the increase in nitric oxide, which was induced by LPS.	[275]
		Triterpenoids	Decreased LPS-induced intracellular ROS	[276]
	*Ganoderma applanatum*	Exopolysaccharides and endopolysaccharides	Non-toxic to embryos. Did not delay or alter hatching, development, and heart rate.	[277]
	*Hericium erinaceus*	Ethanol extracts	Improved locomotion pattern, reduced anxiety, and improved memory by exhibiting anti-acetylcholine esterase activity and antioxidant potential.	[278]
	*Inonotus obliquus*	Polysaccharides	Alleviated oxidative stress, reduced ROS, and reduced apoptosis in developing embryos.	[279]
			Ameliorated the genotoxic effects in UVB-exposed zebrafish by enhancing the expression of DNA repair genes, aiding in normal development	[280]
		2α-hydroxy-inotodiol	Alleviated H_2_O_2_-induced apoptosis in zebrafish head region.	[82]
	*Lentinula edodes*	Ethyl acetate fraction	Reduced prednisolone-induced osteoporosis	[281]
		Wild type and mutant mushroom	Reduced pigmentation in embryos	[282]
		UV-B exposure	Increased the hatching rate and the length of larvae along with an improved anti-inflammatory effect	[283]
	*Pleurotus tuber*-regium	Sclerotium	Inhibited blood vessel formation and subintestinal vessel plexus.	[284]
	*Pleurotus tuber*-regium	Total triterpenes	Reduced changes in the body mass index and lipid accumulation induced by a high-fat diet.	[285]

**Table 4:** The beneficial activities of various mushroom-derived compounds are summarized based on the *Drosophila melanogaster* model used and the mechanism of action.

Model	Mushroom Name	Extract/ Compound	Protective Effects/ Mechanism	References
*Drosophila melanogaster*	*Antrodia camphorata*	Ergosta-7,9(11),22-trien-3β-ol	Improved the life span, motor function, learning, and memory of the AD model. Reduced the biomarkers of microglia activation and inflammation, without affecting lipid peroxidation or catalase and SOD activities.	[288]
	*Ganoderma lucidum*		The formulation (*Panax notoginseng, Panax ginseng,* and gardenoside) aided in memory improvement in the AD model	[289]
	*Hericium erinaceus*	Erinacine A	Extended the lifespan of both male and female Drosophila.	[290]
			Improved survival and locomotion and regulated apoptosis of tert-butyl hydroperoxide-treated ELAV-SCA3tr-Q78 flies.	[291]
	*Lentinus edodes*	Hot water extract	Increased the life span and locomotive activities of male flies but showed early mortality and decreased locomotive activity in female flies.	[292]
	*Lentinus subnudus*		Reduced the levels of acetylcholinesterase and butyrylcholinesterase, ROS and MDA, improved catalase activity, and total thiol levels	[293]
	*Pleurotus ostreatus*	Dried mycelia	Exhibited antigenotoxic activity against mitomycin C mutagen.	[294]
	*Trametes versicolor*	Dried mycelia	Exhibited antigenotoxic activity against mitomycin C mutagen.	[294]

**Table 5:** The beneficial activities of various mushroom-derived compounds are summarized based on the *C. elegans* model used and the mechanism of action.

Model	Mushroom Name	Extract/ Compound	Protective Effects/ Mechanism	References
*C. elegans*	*Auricularia auricula*-judae	Degraded polysaccharides	Extended the lifespan under high sugar stress conditions.	[296]
		Polysaccharides	Extended the lifespan under oxidative stress and improved antioxidant activity	[297]
		Melanin	Extended the lifespan and locomotive properties	[298]
	*Auricularia polytricha*	Ethanol extract	Extended the lifespan and improved pharyngeal pumping rate	[11]
		Polysaccharides	Exhibited antioxidant activity by scavenging free radicals, improving antioxidant enzymes, and reducing the level of ROS during oxidative stress	[299]
			Extended the lifespan, enhanced antioxidant enzymes, and regulated the expression of *skn-1*, *sod-1*, *sod-2*, *sod-3,* and *sir-2.1*	[300]
			Extended the lifespan, enhanced antioxidant enzymes, and regulated the expression of *daf-16* and *skn-1*	[301]
		Acid hydrosylates of polysaccharides	Extended the lifespan, enhanced antioxidant enzymes, and regulated the expression of *daf-16*, *skn-1*, *sir*, *sod-1,* and *sod-2*	[302]
	*Flammulina velutipes*	Polysaccharides	Exhibited anti-ultraviolet activity	[303]
	*Ganoderma lucidum*	Polysaccharides	Extended the lifespan and activate *daf-16* via TIR-1 receptor and the MAPK pathway	[304]
		Water extract	Extended the lifespan and reduced oxidative stress and heavy metal stress. Activation of CR pathway and mTOR/S6K pathway.	[305]
		Water extract and polysaccharides	Extended the lifespan and improved stress resistance by modulating autophagy	[306]
	*Lentinula edodes*	Polysaccharides	Extended the lifespan under heat-induced stress conditions.	[307]
	*Lignosus rhinocerus*	Ethanol extract	Inhibition of Alzheimer’s and Huntington’s diseases.	[114]
		Ethanol, cold water, and hot water extract	Antioxidant and lifespan extension. Upregulated the DAF-16/FOXO pathway.	[308]
